# Assessment of HIV-1 integration in tissues and subsets across infection stages

**DOI:** 10.1172/jci.insight.139783

**Published:** 2020-10-15

**Authors:** Vincent H. Wu, Christopher L. Nobles, Leticia Kuri-Cervantes, Kevin McCormick, John K. Everett, Son Nguyen, Perla M. del Rio Estrada, Mauricio González-Navarro, Fernanda Torres-Ruiz, Santiago Ávila-Ríos, Gustavo Reyes-Terán, Frederic D. Bushman, Michael R. Betts

**Affiliations:** 1 Department of Microbiology and; 2 Institute for Immunology, Perelman School of Medicine, University of Pennsylvania, Philadelphia, Pennsylvania, USA.; 3 Centro de Investigación en Enfermedades Infecciosas, Instituto Nacional de Enfermedades Respiratorias, Mexico City, Mexico.

**Keywords:** AIDS/HIV, Immunology, Cellular immune response, T cells

## Abstract

The integration of HIV DNA into the host genome contributes to lifelong infection in most individuals. Few studies have examined integration in lymphoid tissue, where HIV predominantly persists before and after antiretroviral treatment (ART). Of particular interest is whether integration site distributions differ between infection stages with paired blood and tissue comparisons. Here, we profiled HIV integration site distributions in sorted memory, tissue-resident, and/or follicular helper CD4^+^ T cell subsets from paired blood and lymphoid tissue samples from acute, chronic, and ART-treated individuals. We observed minor differences in the frequency of nonintronic and nondistal intergenic sites, varying with tissue and residency phenotypes during ART. Genomic and epigenetic annotations were generally similar. Clonal expansion of cells marked by identical integration sites was detected, with increased detection in chronic and ART-treated individuals. However, overlap between or within CD4^+^ T cell subsets or tissue compartments was only observed in 8 unique sites of the 3540 sites studied. Together, these findings suggest that shared integration sites between blood and tissue may, depending on the tissue site, be the exception rather than the rule and indicate that additional studies are necessary to fully understand the heterogeneity of tissue-sequestered HIV reservoirs.

## Abstract 2

Deep characterization of HIV integration sites between tissue and blood compartments at multiple infection stages reveals a partitioning of integration sites between and within compartments.

## Introduction

HIV infection remains an important global health issue, despite the advent of antiretroviral therapy (ART). An HIV cure remains elusive, mainly due to integration of viral DNA into the genome of potentially long-lived memory CD4^+^ T cells. This integration of intact HIV-1 proviruses can establish a latent reservoir that is capable of lifelong persistence based on estimated rates of viral decay (1, 2). ART interruption typically leads to viral rebound, indicating that intact proviruses lie dormant during ART treatment but can later be reactivated (3). Increasing our understanding of the characteristics of the HIV-1 reservoir is necessary for the development of a functional cure.

HIV-1 integrates DNA copies of the RNA genome into host cells, predominantly through a coordinated interaction between HIV integrase and host-encoded LEDGF/p75 (4–6). HIV-1 integration sites are found more commonly in active transcription units, a finding which is largely explained by the ability of LEDGF/p75 to tether to these sites (4, 7–10). As HIV-1 integration is not site specific, the host-viral junction site in the genome provides a unique marker of infected cells that allows for the tracking of these cells through integration site analyses. Longitudinal tracking studies have shown an increase in clonally expanded cells during ART, with a selection for cells containing integration sites within cancer-related genes over time (11, 12). These clones are established early in HIV-1 infection and persist after ART treatment (11, 13, 14). However, most integration site studies have been carried out on bulk PBMCs or peripheral blood CD4^+^ T cells, which may not fully recapitulate the complexity of the integrated reservoir in the body for two reasons: (a) the majority of HIV-1–infected CD4^+^ T cells reside in lymphoid tissues (15–17) and (b) CD4^+^ T cells are composed of multiple heterogeneous tissue-sequestered and circulating subsets in vivo (18–22).

CD4^+^ T cells display diverse memory and effector phenotypes, each with different epigenetic and transcriptomic signatures. While many studies have compared various internal proviral sequences across cell subsets (12, 23–27), few studies have compared the integration site landscape between different cell subsets (12). Viral sequence studies generally observed overlapping sequences between cell subsets, but one study has suggested potential compartmentalization based on *nef* sequences between memory cell subsets in 3 of 5 individuals (23). In vivo compartmentalization of CD4^+^ T cells can be seen with tissue-resident memory cells in different contexts, including HIV-1 infection (28, 29). This raises the hypothesis that tissue residency may lead to a different landscape of integration sites between tissue and blood, especially for CD4^+^ T cells that are either tissue resident or have limited egress capabilities. Overall, these studies highlight a gap in our understanding of HIV-1 reservoirs, demonstrating the need to better understand the integration site landscape in the context of tissue/blood compartments and CD4^+^ T cell heterogeneity at different stages of HIV-1 infection.

To address this, we obtained PBMCs and lymph node mononuclear cells (LNMCs) from cervical lymph nodes (CLN) and inguinal lymph nodes (ILN) from HIV-infected untreated acutely infected/chronically infected (acute/chronic) individuals or tonsils from ART-treated individuals. From these, we profiled integration sites within memory, follicular, and resident CD4^+^ T cell subsets to assess the effect of compartmentalization and cellular residency on integration sites. While we observed that HIV-1 integration site targeting characteristics were largely shared in the different compartments, cell subsets, and stages of HIV-1 infection, overlap of integration sites between subsets and compartments was rare, despite the presence of clonally expanded sites. Together, these findings suggest that tissue compartmentalization of the HIV-1 reservoir can occur between different anatomical sites, supporting the need to further understand the dynamics and fate of HIV-infected cells from different cell subsets and tissues.

## Results

### Study design and identification of HIV-1 integration sites.

To assess the effect of compartmentalization and cellular residency on integration site distributions, we sorted LNMCs from ART-treated individuals into 4 CD4^+^ T cell subsets (Supplemental Figure 1; supplemental material available online with this article; https://doi.org/10.1172/jci.insight.139783DS1): (a) CD45RA^+^CCR7^+^ (naive; for individuals T1 and T2 only), (b) HLA-DR^–^CXCR5^hi^PD-1^hi^ (germinal center T follicular helper [GC-Tfh]; HLA-DR^–^ GC-Tfh), (c) non–GC-Tfh memory HLA-DR^–^CD69^+^ (labeled as HLA-DR^–^CD69^+^), and (d) non–GC-Tfh memory HLA-DR^–^CD69^–^ (labeled as HLA-DR^–^CD69^–^). As HLA-DR has been shown to be a marker of T cell activation (30), we sorted HLA-DR^–^ cells to enrich for the latent reservoir in ART-treated individuals as seen in previous studies (31–33). Because the residency marker CD69 is also upregulated by T cells upon activation (34–36), we selectively sorted HLA-DR^–^CD69^+^ cells to focus on resting resident CD4^+^ T cells.

From acutely and chronically infected lymph nodes, we sorted 4 CD4^+^ T cell subsets: (a) CD45RA^+^CCR7^+^ (naive), (b) nonnaive PD-1^hi^CXCR5^hi^ (GC-Tfh), (c) non–GC-Tfh memory CD69^+^ (labeled as CD69^+^), and (d) non–GC-Tfh memory CD69^–^ (labeled as CD69^–^) (Supplemental Figure 2). Given the high viral load and ongoing inflammation in these individuals, we did not exclude HLA-DR^+^CD4^+^ T cells. We did include the CD69^+^ sorting strategy from ART-treated samples to allow analysis of integration sites in resident memory CD4^+^ T cells; however, some of these cells may have been recently activated. Regardless, CD69 expression would still limit tissue egress potential of any recently activated CD69^+^ cells, resulting in potentially unequal distribution of integration sites between tissues and blood.

PBMCs were sorted into memory phenotypes as described in Supplemental Figures 1 and 2. These subsets consisted of (a) CD45RA^+^CCR7^+^ (naive), (b) CD45RA^–^CCR7^+^ (central memory T [Tcm]), (c) CD45RA^–^CCR7^–^ (effector memory T [Tem]), and (d) CD45RA^+^CCR7^–^ (Tem cells reexpressing CD45RA [Temra]) for acute and chronic samples only. For ART-treated individuals, the Tcm and Tem subsets further excluded CD69^+^ cells to select for resting cells. All sort counts are detailed in Supplemental Table 1, while information on individuals is provided in Table 1.

Sorted cells were assayed for integration sites using an established pipeline (37, 38), wherein sonicated adapter-ligated genomic DNA was amplified with a nested PCR using HIV-1 U3 and U5 long terminal repeat primers. Since random fragment lengths are generated during sonication, fragments with different lengths that are associated with integration sites mapping to the same genomic position can be counted as a proxy for the number of infected cells (termed sonic abundance) (38). Even with the limited number of cells (ranging from ~3000 to >400,000 cells per subset), integration site analyses are sufficient to detect clonal CD4^+^ T cell expansions carrying integrated virus (39). We recovered a total of 3540 unique sites and an inferred count of 3973 cells with integration sites across 9 individuals. The breakdown of sites by infection stage, location, and cell subset are shown in Tables 2, 3, and 4, while the entire data set of sites is provided in Supplemental File 1.

### Comparable characteristics in HIV integration sites across infection stage, compartment, and cell subset.

**Figure 1:**
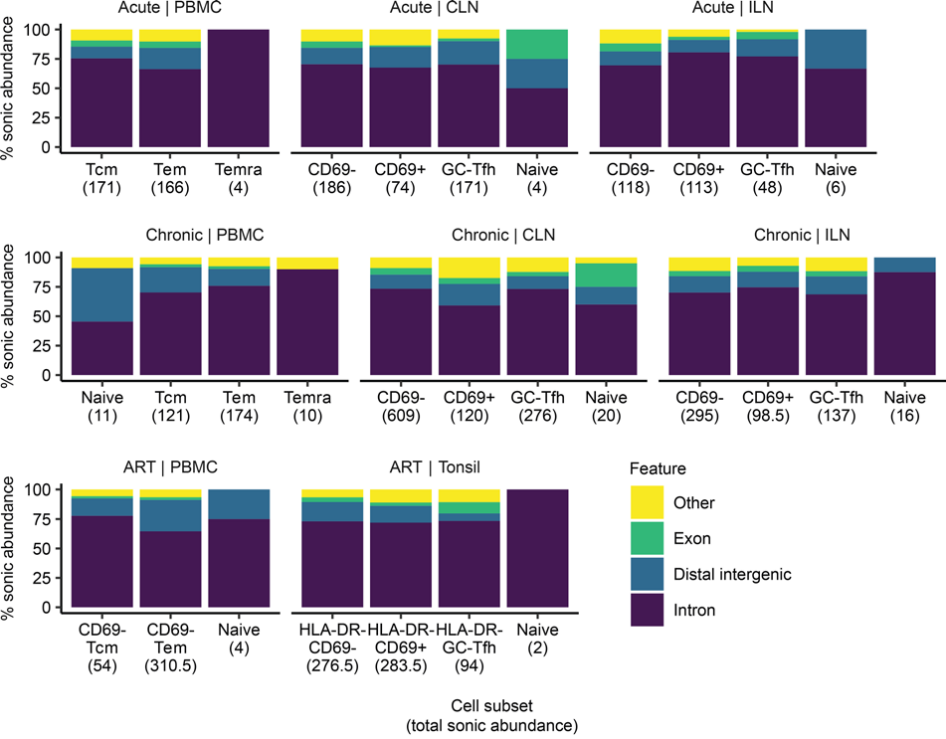
HIV-1 integration sites are primarily found in intronic regions across infection stages, compartments, and cell subsets. For each cell subset, the percentage of total integration sites combined across individuals is displayed for each feature annotation. Integration sites are binned based on cell subset, where inferred cell numbers by sonic abundance are shown in parentheses.

For our initial analysis, we conducted a deep characterization of the genomic characteristics of the integration sites obtained from each donor. Comparable to previous studies (4, 7, 8), the majority of sites were enriched in transcription units, with about 15% being in distal intergenic regions. The majority of the integration sites (71%) were found within introns. When separated by experimental factors (infection stage, compartment, and cell subset), the pattern of favored integration events in transcription units was also observed at similar relative values (Figure 1 and Supplemental Figure 3A). In order to assess any positional biases in chromosomal position, we generated bins after normalizing for chromosomal size (Supplemental Figure 3B). Most integration sites were detected near the end of the chromosome as previously observed (40). While there was no discernible pattern between infection stages, we observed a higher distribution of integration sites at the ends of chromosomes from acute individuals compared with those from chronic or ART individuals (Supplemental Figure 3B). This parallels findings from Haworth et al., where their sites recovered from in vitro cultures had increased frequency at either end of the chromosome compared with sites from 12- to 14-week infections in humanized mice (40).

When grouping our recovered integration sites by infection stage and compartment, we observed similar results with the majority of recovered sites being in intronic regions of transcription units followed by distal intergenic regions. However, there was a trend for an increase in nonintron and nondistal intergenic sites from tissue compartments during ART (Supplemental Figure 3, C and D). When combining these specific annotations (labeled “Other”) into one category and depicting each subset, we observed a significant increase from tissue samples during ART (Supplemental Figure 4A; *P* < 0.05; Wilcoxon rank sum test). This increase was also observed in sites obtained from cellular subsets with a residency phenotype during ART (Supplemental Figure 4B; *P* < 0.01; Wilcoxon rank sum test), suggesting integration sites in resident cells have different integration site profiles or that there are different selective pressures compared with infected nonresident cells during ART. Furthermore, integration sites in distal intergenic regions were enriched in the blood compared with tissue during ART (Supplemental Figure 4A; *P* < 0.05; Wilcoxon rank sum test).

We next pooled the unique transcription unit sites across individuals by infection stage and anatomical compartment and assessed the enrichment of gene ontology annotations using Metascape (41) (Supplemental Figures 5–7). The significant gene ontology terms were similar across the stages, with potentially more enrichment of chromatin modification/organization-related genes in sites identified from lymphoid tissue than those found in blood. Due to the sparsity of our samples, we used a previously published bulk RNA-Seq data set on GC-Tfh and non-Tfh cells from lymph nodes of healthy donors (42) to generate bins of increasing gene expression. Genic integration sites from GC-Tfh cells (independent of HLA-DR) were in genes found in the higher expression bins for the GC-Tfh RNA-Seq data set, but other genic integration sites also followed a similar pattern (Supplemental Figure 8A). This was similarly observed with the non-Tfh cell RNA-Seq data set (Supplemental Figure 8B), suggesting that there was no bias observed for subset-specific integration sites based on gene expression from published RNA-Seq data sets. The overall enrichment of normal cellular pathway and lymphocyte function terms along with the RNA-Seq comparison supports the preferential integration of HIV DNA into genes with higher levels of transcription (43–45).

We then compared genomic annotations of the recovered integration sites, including features such as DNaseI accessibility, CpG counts/density, and GC content (46, 47). Briefly, 3 random control sites were chosen in the human genome for each integration site in the data set to calculate a receiver operating characteristic (ROC) curve. A ROC value of 1 indicates that all actual integration sites in the data have a given feature (or have higher values) compared with all random controls; the opposite is true for a ROC value of 0. A ROC value of 0.5 indicates that this feature is unable to discern between actual and random control sites. For annotations with varied genomic windows, the overall integration site characteristics were comparable across infection stages, compartments, and cell subsets (Supplemental Figure 9A). While there were subtle differences (i.e., GC content within a 1000 bp window being more significantly enriched in the acute and ART-treated samples compared with chronic samples), our findings are consistent with the tendencies of preferential HIV integration into genic regions (4, 44, 46, 48).

Finally, we assessed epigenetic annotations within a 10 kb window of the integration site (±5000 bp). Due to the lack of available annotations specific for each sorted subset, we used epigenetic annotations from bulk CD4^+^ T cells for all comparisons (47). While in general we observed comparable epigenetic annotations, we did identify differences across the different combinations of factors (Supplemental Figure 9B). For instance, H3K79me3 histone methylation was lower in integration sites during ART and chronic states compared with random controls but not during acute infection (Supplemental Figure 9B). H3K79me3 is an important factor for transcriptional regulation that correlates with active gene transcription (49, 50). Further studies would be needed to determine if these sites are depleted in H3K79me3 during ART and chronic states in the specific cell subsets.

### Identification of integration hotspots during multiple stages of HIV infection.

**Figure 2:**
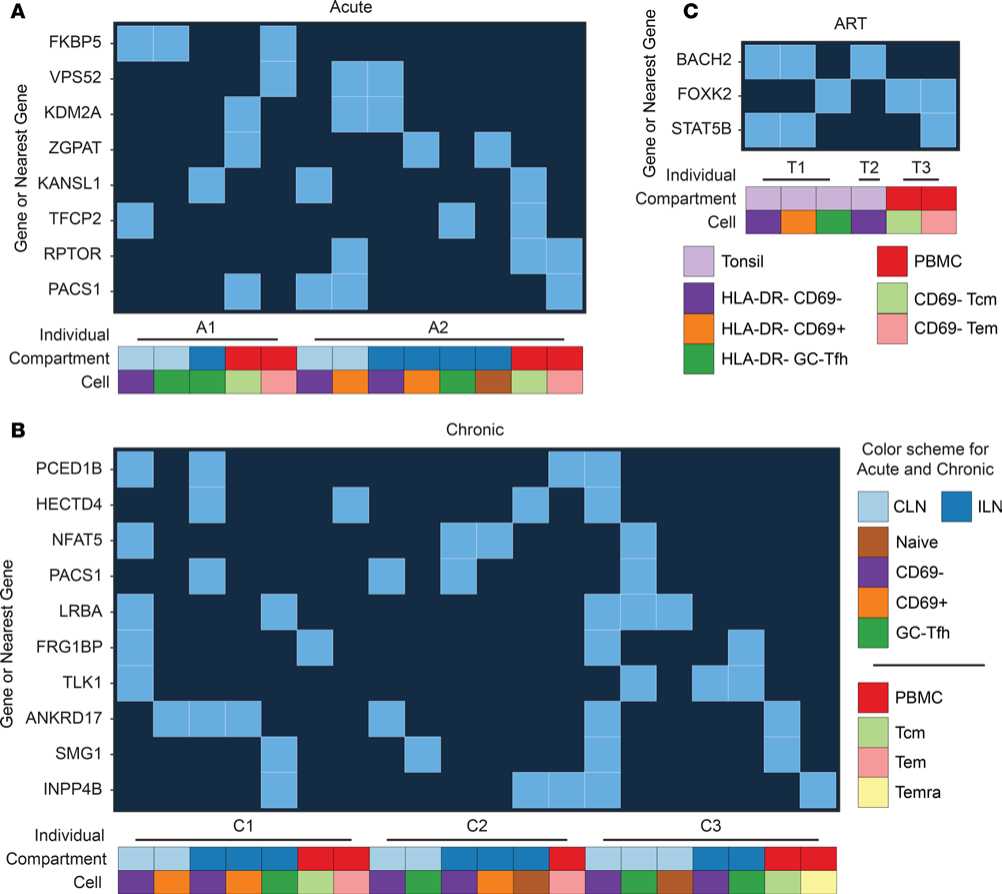
Hotspots of HIV-1 integration across individuals, compartments, and subsets during different infection stages. Integration sites in a gene or within 50 kb of a gene were identified across combinations of individual, compartment, and cell subsets. (**A**) Hotspots identified in 3 or more combinations in acute individuals. (**B**) Hotspots identified in 4 or more combinations in individuals with chronic HIV-1 infection. (**C**) Hotspots identified in 3 or more combinations in ART-treated individuals.

We next identified genes commonly targeted for integration during different stages of HIV infection. We filtered the integration sites for genes (or nearest genes within 50 kb) present in 3 or more combinations of individuals/cell subsets/compartments for acute and ART-treated individuals (Figure 2, A and C). We raised this threshold to 4 or more combinations for chronic individuals due to the larger number of genes (Figure 2B).

For acute and chronic infections (Figure 2, A and B, respectively), we identified numerous hotspots. Integrations in or near *PACS1* and *ANKRD17* were the most common hotspot in acute and chronic data sets, respectively. Furthermore, we detected integrations within or near *STAT5B* and *FOXK2* genes during acute and/or chronic states, lending support to the notion that clonally expanded sites during ART treatment can be established early during infection (13, 14, 51). We also assessed pairwise enrichment of genes between infection stages and identified genes trending toward enrichment in different infection states (Supplemental Tables 2–4). We did not assess statistical significance due to the limited power of our sample size.

In ART-treated individuals, we observed a number of genes classically associated with integration, including *STAT5B*, *FOXK2*, *BACH2*, and *TET2/TET2-AS1* (13, 51, 52), across different subjects, tissues, and CD4^+^ T cell subsets. However, none of these integration-associated genes were found in every individual or within all of the assessed cell subsets within an individual. In the HLA-DR^–^CD69^–^ population from tonsils of individual T1, we detected a *TET2* (and *TET2-AS1*) integration in intron 2, which is of clinical interest given a recent report on the in vivo expansion of a CAR T cell with an integrated lentiviral vector in *TET2* (47, 52). Together, our data support previous studies documenting the enrichment of certain sites during ART treatment, but highlight the differential complexity of the integrated reservoir within each individual. We did not find any single specific gene preferentially associated with integration across all individuals or tissues.

### Detection of clonal expansion across multiple stages of infection.

**Figure 3:**
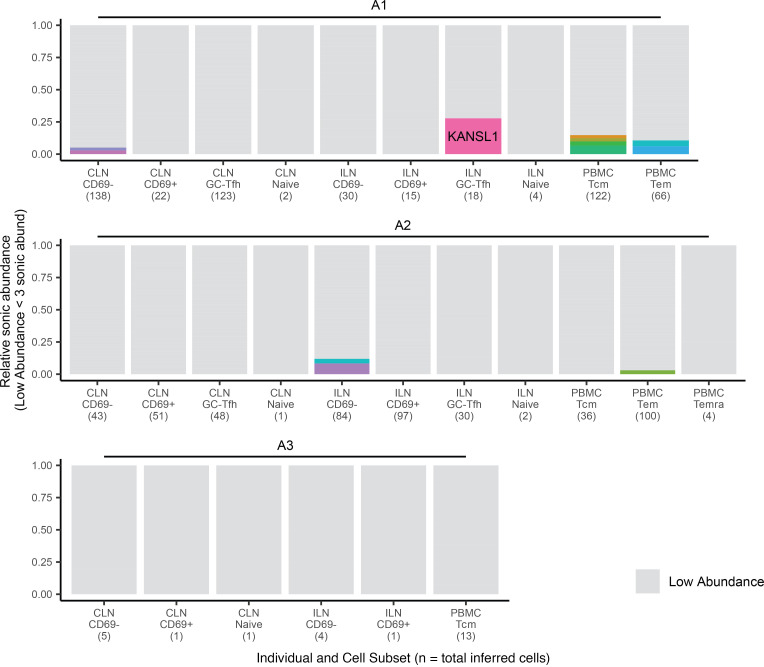
Identification of clonal expansion from individuals during acute infection. Relative sonic abundance for each integration site is shown for each acutely infected individual. Sites with color indicate that the site is clonal (defined as a sonic abundance ≥3 for acute infection). Different colors indicate different integration sites across an individual (i.e., two different integration sites in the same gene with clonal expansion would have different colors). Clones consisting of 10% or more of the total relative sonic abundance are labeled with the integrated gene or nearest gene. Genes within 50 kb of the integration site are denoted with “+,” while genes that are more then 50 kb from the integration site are demoted with “#.” The number of inferred total cells is indicated parenthetically on the *x* axis.

**Figure 4:**
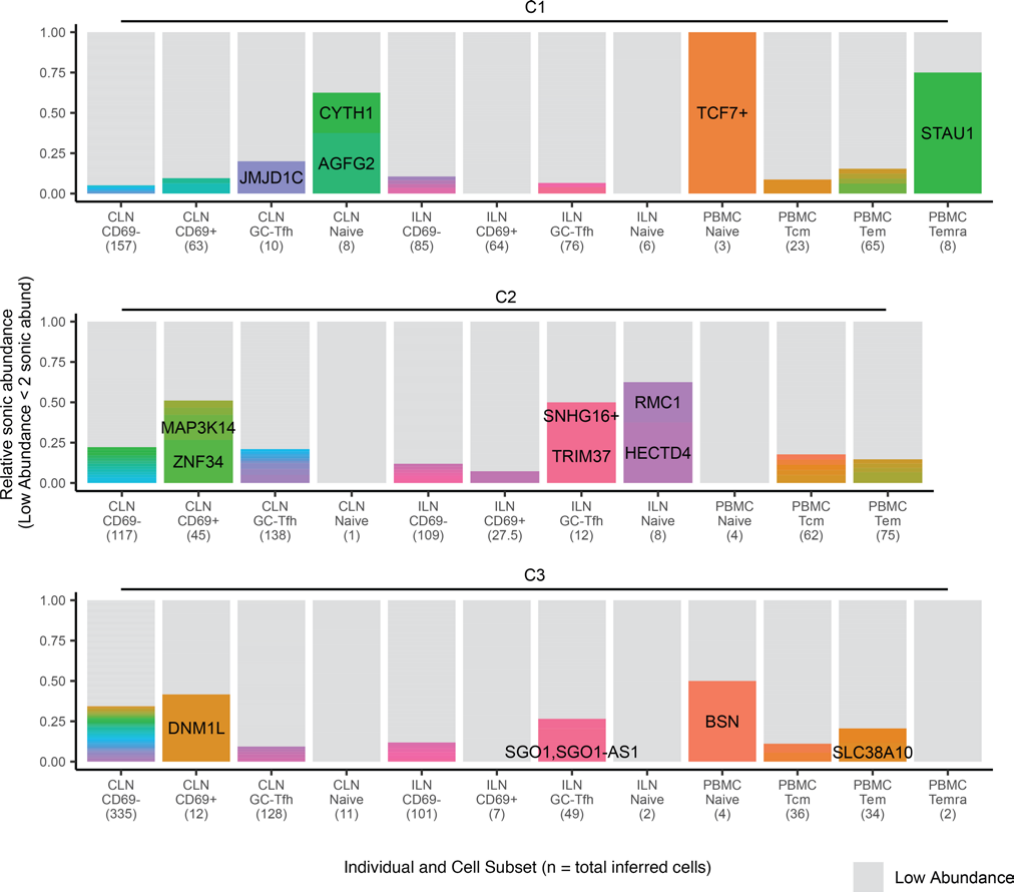
Identification of clonal expansion from individuals during chronic infection. Relative sonic abundance for each integration site is shown for each individual. Sites with color indicate that the site is clonal (defined as a sonic abundance ≥2 for chronic infection). Different colors indicate different integration sites across an individual (i.e., two different integration sites in the same gene with clonal expansion would have different colors). Clones consisting of 10% or more of the total relative sonic abundance are labeled with the integrated gene or nearest gene. Symbols appear after gene names as detailed in the legend for Figure 3.

**Figure 5:**
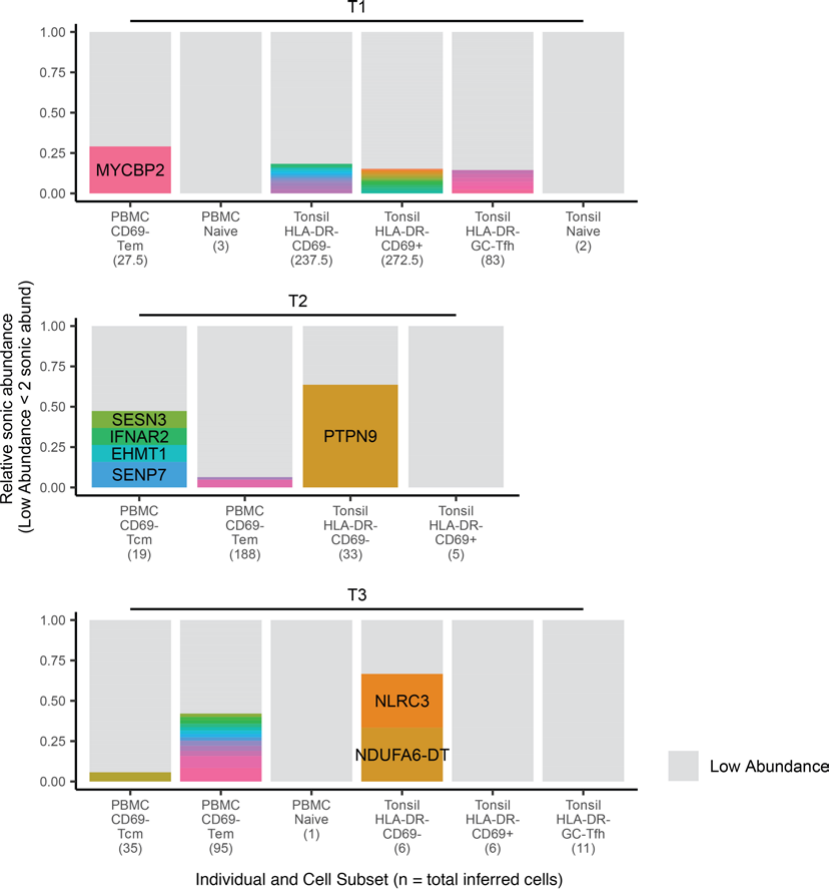
Identification of clonal expansion from ART-treated individuals. Relative sonic abundance for each integration site is shown for each individual. Sites with color indicate that the site is clonal (defined as a sonic abundance ≥2 for ART-treated individuals). Different colors indicate different integration sites across an individual (i.e., two different integration sites in the same gene with clonal expansion would have different colors). Clones consisting of 10% or more of the total relative sonic abundance are labeled with the integrated gene or nearest gene. Symbols appear after gene names as detailed in the legend for Figure 3.

Clonal expansion in peripheral blood has been documented previously during ART treatment (13, 51) as well as acute and chronic infection (14) but has not been compared between blood and lymphoid tissue at multiple stages of infection. Using sonic abundance, we could identify separate fragments with the same integration site originating from clonally expanded cells with the same integration site in all 3 stages of infection (Supplemental File 1 and Figures 3, 4, and 5). To identify cell clones during acute infection (Figure 3), we used a sonic abundance threshold of 3 to ensure the detection of at least 2 infected cells, as cells undergoing mitosis during acute infection may die (14). We found limited examples of clonal expansion during acute infection, with the most dominant clone (by absolute sonic abundance) integrated in *TUBGCP6* in the PBMC Tcm subset from individual A1 (Figure 3). For both the chronic and ART-treated samples, the sonic abundance threshold was set to 2. In chronic samples, we detected higher levels of clonal expansion, with some clones representing 10% or more of the total relative abundance in a cell subset (Figure 4). The most dominant clone detected in a chronic individual was integrated in *ZNF34* within the CD69^+^ subset in CLN from individual C2. During ART treatment, some clones also represented 10% or more of the total infected cell population detected in a given cell subset, similar to our observations for chronic individuals (Figure 5). The most dominant clone, by absolute sonic abundance, contained an integrated provirus in *PTPN9,* within the HLA-DR^–^CD69^–^ subset in the tonsil from individual T2. Overall, these results indicate that clonal expansion can be detected in tissues early in infection as well as during chronic infection and ART treatment.

Within the subset and infection stage resolution across our cohort, we asked if any particular subsets (pooled across tissue sites) were enriched for clones. As detailed in Supplemental Table 5, most subsets contained clones. In acute individuals, Tcm cells had the highest percentage, with 2.78% of detected unique sites being clonal. The maximum values were 9.54% (CD69^–^ from tissues) from chronic individuals and 7.56% (HLA-DR^–^CD69^–^ from PBMCs) from ART-treated individuals when considering only subsets in which we recovered more than 100 unique sites. Furthermore, clonal proportions significantly differed between infection stages (Supplemental Figure 10). There was also a trend toward higher clonality in nonresident phenotypes compared with resident phenotypes in chronic and ART-treated individuals, but this did not reach statistical significance, likely due to limited sample size. Together, these data suggest that the proportion of clonal sites increases over time during the course of infection and with the installment of ART and highlights the need to further explore tissue-resident phenotypes in the context of HIV-1 reservoirs.

### Limited overlap detected between cell subsets and compartments.

Recent reports have documented overlap of proviral sequences across compartments and across subsets. We thus examined whether there was overlap of integration sites within individuals between different T cell subsets. Since integration sites can serve as a unique barcode of an infected cell, we used integration site data to examine if infected cells after cell division have trafficked and/or differentiated.

**Figure 6:**
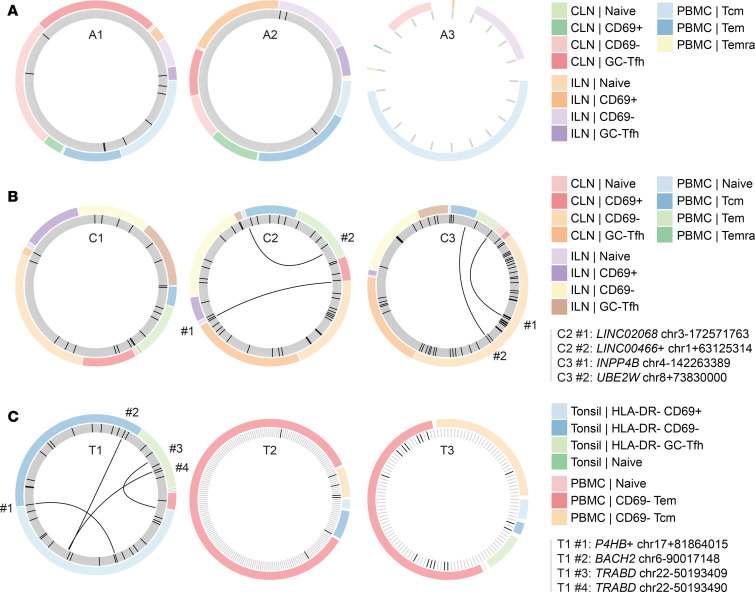
Limited overlap of integration sites detected within cell subsets and compartments. For (**A**) acute, (**B**) chronic, and (**C**) ART-treated states, each plot represents an individual. Each spoke on the inner wheel represents a unique integration site. Spokes with a darker color represent clonally expanded sites. The outer wheel represents the compartment and subset where the integration site was detected. Connecting lines indicate that the same integration site was detected in a different cell subset and is labeled with the gene (or nearest gene) along with genomic location. Symbols appear after gene names as detailed in the legend for Figure 3.

For acute infection, we detected no overlap in any of the individuals (Figure 6A), likely due to the rampant viral replication and death/turnover of infected cells. Despite the depth of our analysis, we only detected a few overlaps of integration sites between data from T cell subsets or anatomic sites in 2 chronically infected individuals (Figure 6B). These included a clone in *LINC02068* shared between ILN (naive) and CLN (CD69^–^), a clone near *LINC00466* in PBMCs shared between naive and Tem cells, a clone in *UBE2W* shared between CLN (CD69^–^) and PBMCs (Tcm), and a clone in *INPP4B* shared between CLN (CD69^–^) and PBMCs (Temra).

We only detected overlap of integration sites between subsets or tissue in 1 of the 3 ART-treated individuals (Figure 6C). These included a *BACH2* clone shared between tonsil HLA-DR^–^CD69^+^ and HLA-DR^–^CD69^–^CD4^+^ T cells, 2 separate *TRABD* clones between tonsil HLA-DR^–^ GC-Tfh and HLA-DR^–^CD69^+^CD4^+^ T cells, and an expanded clone near *P4HB* between tonsil HLA-DR^–^CD69^–^ and HLA-DR^–^CD69^+^CD4^+^ T cells. No overlaps were detected between peripheral blood CD4^+^ T cells and tonsil cells in any of the 3 ART-treated individuals, despite the detection of clonal expansion (Figure 6C).

We assessed partitioning of clones in different anatomical sites by comparing our observations to results of computational simulations. We assumed that clones could be shared randomly between compartments and/or subsets. For each individual, we pooled all the sites according to their sonic abundance and then randomly sampled the pool based on the original sample size recovered in a given cell subset. The number of overlaps were counted across 10,000 simulations. Our simulations indicate that considerable overlap is expected in all pairs of samples tested (Supplemental Figure 11), in contrast to our observations of limited overlap. Thus, our integration site data set was not dominated by expanded clones that were common to multiple compartments, though it will be valuable to challenge this conclusion in future studies with deeper sampling over more subjects.

## Discussion

Our current understanding of the integrated HIV-1 reservoir is largely derived from studies of the peripheral blood on bulk CD4^+^ T cells, with the assumption that these represent infected CD4^+^ T cells in lymphoid tissues. While some infected CD4^+^ T cells may traffic between tissues and blood, it is unclear to what degree this occurs. Moreover, some memory CD4^+^ T cells can establish residence in tissues and thus may not be well represented in the circulation. For example, Tfh cells, a population known to be a key HIV-1 reservoir, are mostly anatomically restricted to lymphoid tissues. These anatomical and subset characteristics of CD4^+^ T cells raise the question of whether bulk peripheral blood CD4^+^ T cell populations provide an accurate representation of the HIV-1 integration landscape throughout the body at multiple stages of infection. To address this gap, we examined integration site distributions in acute, chronic, and ART-treated HIV-1 individuals at compartment and subset resolutions. We found comparable characteristics of integration site distributions, where most sites were found in transcription units. The CD4^+^ T cell subset, tissue of origin, or the infection stage had at most slight effects on the initial integration site selection based on comparison to genomic and epigenetic annotations. Previously identified genes that are commonly detected in infected cells were also found among our data set. As expected, clonal expansion was found in most compartments and was more prominent in chronic and ART-treated individuals. Despite these expanded clones, we detected little overlap between subsets and compartments.

The general observation of similar characteristics of HIV-1 integration across subset and compartment supports previous studies documenting the tendencies of HIV-1 integration. While it is known for HIV-1 to prefer sites of active transcription, our observed lack of noticeable transcriptional activity bias by subset could potentially be explained by similar global transcriptomic profiles of CD4^+^ T cell subsets, irrespective of known differentially expressed subset-defining genes. Despite largely similar characteristics, we were still able to detect some differences in our comparisons. We observed a corresponding increase in frequencies of nonintronic and nondistal-intergenic sites during ART in tissue and resident subsets, suggesting that infected resident cells might have subtle differences compared with nonresident infected cells. Furthermore, the trend in increased detection of clonality in nonresident cell subsets requires more sampling to better understand how residency phenotype may impact HIV-1 reservoirs.

Recent studies have investigated the HIV-1 integration site landscape in bulk CD4^+^ T cells or LNMCs from lymphoid tissue, finding integration site overlaps between tissue compartments in ART-treated individuals (39, 53). This suggests that trafficking of infected cells may occur between compartments over long-term ART treatment. Other groups have observed identical internal proviral sequences between lymphoid tissue and peripheral blood (24, 27, 39, 53–55). However, the existence of identical proviral sequences between compartments does not indicate clonality, as different cells can harbor the same proviral sequence integrated at different sites in the host genome (39).

The limited overlap between integration sites in different subset and compartments in our data set has potential ramifications. With limited ongoing viral replication in lymphoid tissue during ART (53) and the low likelihood of the same integration site occurring from two separate cellular infection events, the emergence of identical sites in different compartments would require proliferation of the infected cell followed by migration and/or differentiation. This lack of integration site overlap suggests that migration and/or differentiation may impart a fitness cost (whether by immune clearance from viral reactivation, disruption of required cellular/differentiation programs due to integration, or other means). This scenario is supported by the observation that the infection of resting memory CD4^+^ T cells likely stems from an infection event during an activated state prior to the transition to a quiescent state, thus escaping elimination (56). Any exit from quiescence could lead to the presentation of viral antigens and subsequent clearance, resulting in the general maintenance of segregation. The observation of shared proviral sequences from before ART treatment (27) and our limited observation of integration site overlap supports the notion of a genetic bottleneck (likely from immune pressure prior to ART, ref. 39).

Insertional mutagenesis affecting specific cellular genes may be linked to cellular proliferation. Some of the overlapping genes that we detected have been documented for their roles in oncogenesis. *LINC00466* has been implicated as a potential factor in promoting lung adenocarcinoma (57). *BACH2* has known tumor immunosuppressive capacity (58), and *P4HB* upregulation may be involved with glioblastoma multiforme (59) as noticed for integration sites recovered from the ART-treated individuals, thus suggesting that oncogenic genes may play a role in the overlap. Strikingly, these limited overlaps were all within the same compartment or involved the nonresident subsets (i.e., all subsets except tissue CD69^+^ or GC-Tfh phenotypes), suggesting that migration of infected clones through lymphatic tissues does occur. The dilution of a given infected clone among the vast network of lymphatics and vasculature could explain the small degree of sharing among the nonresident subsets.

Recent studies reported overlap in bulk CD4^+^ T cells between tonsils and peripheral blood in ART-treated individuals (39, 53), but our data highlighted the lack of overlap in our subjects. The statistical concern of the “unseen species problem” (where the overlapping clones might have been undetected given the size of the pool of infected cells) is always present for these types of studies, especially with our samples from peripheral blood during ART. However, clonal expansion was still detectable from both compartments in our data set. The discrepancy between our findings and these previous studies can potentially be explained by two hypotheses. First, the duration of ART treatment may play a role in the emergence of sharing between blood and tissues. Our study cohort was sampled at a generally shorter duration of ART (1.4, 7.8, and 3.2 years) compared with previous studies (5.7, 13, and <15.8 years) (53). Time may be needed under suppressive therapy to allow cells to differentiate after antigenic stimulation or equilibrate between the subsets and/or compartments. Second, the overlaps between blood and tonsil may exist in the HLA-DR^+^-activated subset, as we specifically examined resting HLA-DR^–^ cells for tonsil samples. A recent study showed that there was an increase in infected HLA-DR^+^ cells over time during ART (26), suggesting that these two hypotheses are not mutually exclusive. Both HLA-DR^+^ and HLA-DR^–^ cells are capable of harboring intact proviruses and in some cases have identical p6-RT sequences (26). However, integration site distributions were not assessed, thus leaving the question of true overlap between these subsets unresolved. The presence of HLA-DR and the likelihood of an activation state may suggest the ability for cells to traffic between compartments.

The limited overlap of integration sites reported here in the HIV-1 reservoir has consequences for cure strategies. Latency reversal agents to date have generally been unable to “shock” the entire reservoir, thus limiting the effectiveness of viral elimination. A recent study has demonstrated differential effects in activating HIV-1 RNA transcription via latency reversal agents in diverse CD4^+^ T cell memory subsets from blood (60). These previous findings, combined with the differences we observed based on residency phenotypes, highlight the need to better understand the HIV-1 reservoir at an increased subset resolution.

Our study has several limitations, with the primary limitation being our sample size. Due to the large size of the HIV-1 reservoir and the possibility of single sites being actual clones, more studies with a variety of lymphoid tissues, including the gut-associated lymphoid tissue (GALT), are needed from individuals in different cohorts to validate our findings. While we did profile from 2 lymph nodes that are distal to each other, this does not preclude the possibility of clonally expanded cells being in the remaining hundreds of other structures distributed throughout the body. However, these samples, especially the multiple paired lymph nodes are difficult to acquire and costly to sequence at the cell subset resolution. Another limitation is the lack of dual integration site and viral DNA sequencing at the time of our study, which was difficult with the number of cells procured from each subset. As studies have documented the existence of replication competent viruses in clonally expanded cells (25, 39, 61), the understanding of which cells in our study have replication competent viruses would add additional resolution. New techniques for paired integration site and proviral profiling could be used for further studies (25, 39). The identification of integration sites, however, is sufficient for determining clonal expansion in infected cells (39).

Overall, our data set provides a resource to the HIV community, as it is the first to our knowledge to profile integration sites from acute, chronic, and ART-treated individuals with paired tissue and blood comparisons at a subset resolution. This resource will be valuable for both the HIV-1 reservoir field as well as for clinical methods using retroviral vectors. Our data highlight the need to better understand the reservoir — in terms of specific subreservoirs that may need to be differentially targeted with a focus on the residency status of infected cells.

## Methods

### Samples.

For acute and chronic infection studies, peripheral blood and tissue samples (CLN and ILN) were simultaneously obtained from HIV-1^+^ individuals prior to the introduction of ART (*n* = 3 for acute and *n* = 3 for chronic). For ART-treated studies, peripheral blood and tonsil samples were simultaneously obtained at the same time point from HIV-1^+^ individuals undergoing ART treatment (*n* = 3). Donor information is summarized in Table 1.

PBMCs and LNMCs were prepared as previously described (62). All cells were cryopreserved before being shipped on dry ice for experimental studies.

### CD4^+^ T cell subset sorting.

PBMCs and LNMCs were thawed and suspended at 2 million cells/mL in complete RPMI medium supplemented with 10% FBS, 1% L-glutamine, and 1% penicillin/streptomycin (R10). Cells were rested overnight in a humidified incubator with 5% CO_2_ at 37°C, and RNase-free DNase I (10 units for every mL media used; MilliporeSigma) was added to reduce cell clumping for sorting. After overnight rests, cells were washed with PBS prior to extracellular staining of cellular markers for FACS on the same day.

Cells were first stained with CCR7 APC-Cy7 (G043H7, BioLegend, 353211) for 10 minutes in a humidified incubator with 5% CO_2_ at 37°C. LIVE/DEAD Fixable Aqua (Thermo Fisher Scientific) was then added to the cells for a 10-minute incubation at room temperature. Cells were stained with a master mix of monoclonal antibodies with fluorescent conjugates (antibodies and clones are listed below) for 20 minutes at room temperature. Samples with greater than or equal to 15 × 10^6^ cells were subjected to 2× of the titration volumes for all staining steps. Cells were then washed with PBS before being resuspended in 350 μl phenol red–free RPMI if samples had greater than or equal to 15 million cells. Otherwise, cells were resuspended in 250 μl phenol red–free RPMI. Prior to FACS, the cells were subjected through a 35 μm nylon mesh strainer-top tube (Thermo Fisher Scientific) to reduce cell clumping. All samples were sorted with a FACS Aria II SORP (BD) situated in a biosafety cabinet for biohazardous samples using standard methodology. The gating strategy is described in Supplemental Figures 1 and 2. Individual T3 had a further CD32^–^ gate on cells from tonsil, as this sample’s sort was done from a previous study (63). Cells were sorted into the 1.5 mL DNA LoBind tubes (Eppendorf) prefilled with 200–300 μl of a solution containing 1 part FBS and 1 part phenol red–free RPMI or PBS.

Sorted cells were pelleted in a centrifuge at 3000*g* for 5 minutes, and supernatants were discarded. Cell pellets were resuspended in 200 μl PBS and snap frozen on dry ice before storing at –80°C.

### Antibodies.

The following monoclonal antibodies were used for FACS: CCR7 APC-Cy7 (G043H7, Biolegend, 353211), CD69 PE (FN50, BD, 555531), CD3 APC R700 (UCHT1, BD, 565119), PD-1 BV421 (EH12.2H7, Biolegend, 329920), HLA-DR BV605 (G46-6, BD, 562845), CXCR5 BB515 (RF8B2, BD, 564624), CD14 PE-Cy5 (61D3, abcam, ab25395), CD16 PE-Cy5 (3G8, Biolegend, 3012010), CD19 PE-Cy5 (HIB19, Biolegend, 302210), CD45RA PE CF594 (HI100, BD, 562298), CD8 PE-Cy5.5 (RPA-T8, eBioscience, 35-0088-42), and CD4 PE Cy7 (RPA-T4, Biolegend, 300512).

### Integration site profiling.

Sorted cells were thawed for DNA extraction using the DNeasy Blood & Tissue Kit (Qiagen) using standard protocols. The final eluate was reeluted through the column to improve DNA yields, as recommended in the manufacturer’s protocol. 100 μl of the DNA eluate was supplemented with 30 μl molecular-grade water (mH_2_O) to proceed with library preparation as previously published (37, 38). Briefly, genomic DNA was sonicated using a Covaris M220 unit (peak power: 50 W, duty factor: 5%, cycles/burst: 200, treatment time: 60 seconds, water temperature: 25°C) to generate approximately 800–900 bp fragments. A genomic uninfected DNA control (purified from 293T cells; Bushman Lab) was added during the sonication stage. Fragmented DNA was cleaned using AMPure XP beads (Beckman Coulter) (ratio of 0.7 beads/1 DNA volume) and resuspended in 40 μl mH_2_O.

DNA ends were repaired using NEBNext Ultra End Repair/dA-Tailing Module (New England Biolabs) and incubated at 20°C for 30 minutes, 65°C for 30 minutes, and rested at 4°C indefinitely. A no-template control of mH_2_O was added during the end-repair stage. End-repaired fragmented DNA was then ligated with unique linkers using the NEBNext Ultra Ligation Module (New England Biolabs) for 16 hours at 16°C and rested at 4°C indefinitely (38). The resulting product was then bead purified using AMPure XP beads (0.7 beads/1 DNA volume) and eluted in 60 μl of mH_2_O.

The first round of nested PCR (PCR1) was performed in replicates of 4 per sample using the Advantage 2 PCR kit (Takara Bio). 15 μl DNA from the previous step was used for each replicate. U3 and U5 primers targeting the HIV-1 LTR region primers and unique linker primers were added at a final concentration of 300 nM each. Final volume was adjusted to 25 μl per replicate with mH_2_O. Thermocycler settings were set for 1 cycle of 95°C for 1 minute; a linear amplification of 5 cycles of 95°C for 30 seconds plus 70°C for 1 minute and 30 seconds; a log amplification of 20 cycles of 95°C for 30 seconds plus 67°C for 1 minute and 30 seconds; a final extension cycle of 70°C for 4 minutes; and an indefinite hold at 4°C. 5 μl PCR1 product was run on a 1% agarose-TAE gel to check for smears.

The second round of nested PCR (PCR2) was performed on 2 μl PCR1 product for each replicate. Samples at this stage were separated into 2 different reactions, one containing U3 primers and the other containing U5 primers. All LTR primers contained sequencing adaptors as well as a unique barcode with the goal of all samples (including replicates) having a unique LTR-associated barcode and linker combination. PCR2 linker primers and U3 or U5 primers were added at a final concentration of 300 nM each. No internal fragment blocking oligo was added. Final volume was adjusted to 25 μl with mH_2_O. Thermocycler settings were set for 1 cycle of 95°C for 1 minute; a linear amplification of 5 cycles of 95°C for 30 seconds plus 70°C for 1 minute and 30 seconds; a log amplification of 15 cycles of 95°C for 30 seconds plus 67°C for 1 minute and 30 seconds; a final extension cycle of 70°C for 4 minutes; and an indefinite hold at 4°C. 5 μl PCR2 product was run on a 1% agarose-TAE gel to check for smears.

All PCR2 replicates were pooled together (U3 and U5 products are kept separate). PCR2 replicate pools were bead purified with AMPure XP beads (0.7 beads/1 DNA volume) and eluted in 40 μl mH_2_O. Libraries were then assessed for molarity of readable amplicons using quantitative PCR with the 2X MasterMix, ROX Low kit (Roche). Average fragment size was determined with an Agilent 2200 TapeStation using a High Sensitivity D1000 ScreenTape Assay.

Samples were pooled using the calculated readable molarity to attempt to equalize molarities across samples by using the Microsoft Excel Solver add-in. 1 μl each of uninfected and no-template controls were added to this pool. Bead purification with AMPure XP beads (0.7 beads/1 DNA volume) was performed as needed to get a final molarity of approximately 2 nM or greater. Final readable molarity concentrations were determined for the pooled library in addition to an average fragment size determination. Samples were then prepared for paired-end sequencing using a 300 cycle MiSeq v2 Reagent Kit (Illumina) with 30% PhiX spike-in and custom primers on the MiSeq platform (Illumina).

### Primers.

All primers are displayed in a 5′–3′ direction and were manufactured by Integrated DNA Technologies. Linker-associated primers and barcodes are from a previous study (38). The following primers were used: HIV-1 PCR1 (U3): CCCTGGCCCTGGTGTGTAGTTCTG; HIV-1 PCR1 (U5), GAACCCACTGCTTAAGCCTCAATAAAG; HIV-1 PCR2 (U3), CAAGCAGAAGACGGCATACGAGAT**BARCODE**AGTCAGTCAGCCCAGGGAAGTAGCCTTGTGTGTGGT; HIV-1 PCR2 (U5), CAAGCAGAAGACGGCATACGAGAT**BARCODE**AGTCAGTCAGCCCAAGTAGTGTGTGCCCGTCTGTTG; sequencing R1, ATCTACACCAGGACTGACGCTATGGTAATTGT; sequencing index (U3), CAAGGCTACTTCCCTGGGCTGACTGACT; sequencing index (U5), AGTCAGTCAGCCCAGGGAAGTAGCCTTG; sequencing R2 (U3), GGGCACACACTACTTGGGCTGACTGACT; and sequencing R2 (U5), AGTCAGTCAGCCCAAGTAGTGTGTGCCC.

### Read to site mapping and abundance scoring.

The processing of raw reads to final integration site counts was done using the cHIVa pipeline (https://github.com/cnobles/chiva; branch, master; commit ID, d43d01ebc577bb626dbdcda2eddefc9dc7387469), which is a streamlined and updated version of the original INSPIIRED pipeline (https://github.com/BushmanLab/INSPIIRED; branch, master; commit ID, 35e4b0b06182e3dcdb15f2abb6dbaab45b0ac225) (37). Briefly, raw bcl files from sequencing were converted into fastq files using bcl2fastq2 (Illumina). The fastq files are demultiplexed, trimmed, filtered, consolidated, aligned, and further processed with crossover filtering to get human (hg38) and viral integration site junctions by sample. Sonic abundance was then calculated as previously described by counting the unique fragment lengths for a given integration site to infer the number of infected cells containing the site of interest (37, 38). Since U3 and U5 were multiplexed together, the average of U3 and U5 sonic abundances for each original PCR replicate was used if there were both U3 and U5 reads detected with the same integration site. Additional R code (https://github.com/wuv21/intsite_analysis; branch, master; commit ID, 2d770df836b556cb841caef0d1124c1aeed5c058) was used to process and combine data from different runs for graphs and summary information. The ChIPSeeker package in R was used to annotate sites based on genomic location (64). Demultiplexed sequencing files are deposited in the Sequence Read Archive database (accession PRJNA655671).

### RNA-Seq analysis.

Bins of genes with increasing transcriptional activity were derived from published RNA-Seq data sets from GEO series GSE130793 (42). GC-Tfh samples (GSM3753986, GSM3753990, and GSM3753994) and non-Tfh (GSM3753987, GSM3753991, and GSM3753995) from lymph nodes were used. The raw counts were read using the R statistical language with the DESeq2 package (65). After normalization and scaling with default parameters, the transcriptional levels for each gene were then placed into 10 equal bins. The levels were determined by the samples within each subset (GC-Tfh or non-Tfh). Integration sites were then filtered from our data set to select for those residing in a gene. Sites were pooled together based on subset (as indicated in Supplemental Figure 8). Since genes may overlap, we included all of the overlapping genes in the comparison. The proportion of our recovered genic integration sites within each transcriptional bin was counted and plotted.

### Genomic and epigenetic annotation heatmaps.

Integration sites were grouped based on criteria of interest. Groups of sites were only included if there were 30 or more sites in preparation for site annotations as previously detailed (46–48). Briefly, 3 matched random control (MRC) sites were chosen for each integration site in the filtered data set. The actual and MRC sites were then marked for different genomic and CD4^+^ T cell epigenetic annotations in order to compute a ROC curve. Significance for the proportions between actual integration sites and MRC sites was found using a χ^2^ test.

### Pooled overlap sampling.

For each individual, all integration sites were pooled based on their sonic abundance. The sites were then randomly sampled by the original sonic abundance of a given cell subset to preserve our original sequencing depth and breadth. If a site was found in 2 or more of these simulated cell subsets, the site would be recorded as being a simulated overlap. This simulation was repeated 10,000 times, and the number of simulated overlaps detected was plotted as a cumulative distribution frequency plot.

### Statistics.

Statistical analyses are detailed in the Methods and figures legends and are consolidated here. Differences between genic annotations were assessed using a Wilcoxon rank sum test. For the genomic/epigenetic heatmaps, significance between actual integration sites and MRCs was assessed using a χ^2^ test. Clonal proportions by residency phenotype and by stage were assessed using a Wilcoxon rank sum test. *P* values below 0.05 were deemed significant.

### Study approval.

All donors were recruited by the Department of Infectious Diseases at the National Institute for Respiratory Diseases (INER-CIENI) in Mexico City, Mexico. All donors provided written informed consent in compliance with protocols set forth by the INER-CIENI Ethics Committee and the Institutional Review Board at the University of Pennsylvania. Protocols were approved by the Institutional Review Boards at the University of Pennsylvania (no. 809316) and Comité de Ciencia y Bioética en Investigación (Committee for Science and Bioethics in Research) from INER (Mexico City, Mexico; study no. B03-16).

## Author contributions

VHW, FDB, and MRB conceptualized experiments, analyzed data, and wrote the manuscript. PMDRE, MGN, SAR, and GRT provided the cells for the study. VHW and SN conducted the cell culture before and after sorting. LKC performed the flow cytometry sorting. FTR performed the surgeries to acquire inguinal lymph nodes. VHW and SN performed the integration site assay. VHW, CLN, KM, and JKE analyzed the sequencing data and performed downstream computational analyses.

## Supplementary Material

Supplemental data

Supplemental Data Set 1

Supplemental Tables 1-5

## Figures and Tables

**Table 4:**
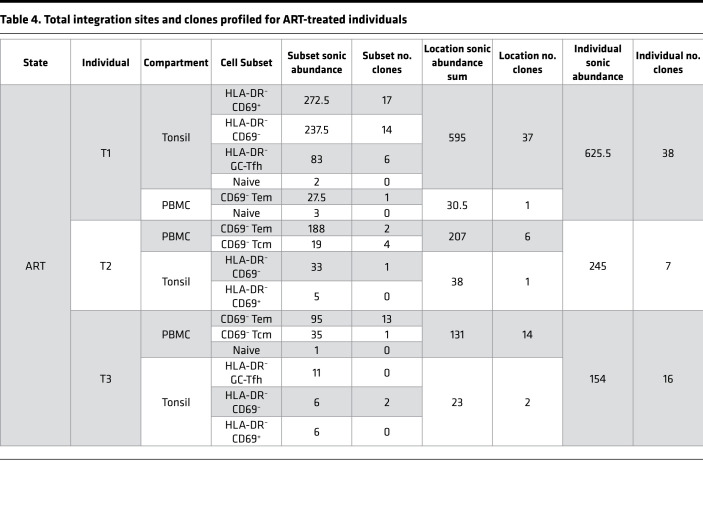
Total integration sites and clones profiled for ART-treated individuals

**Table 3:**
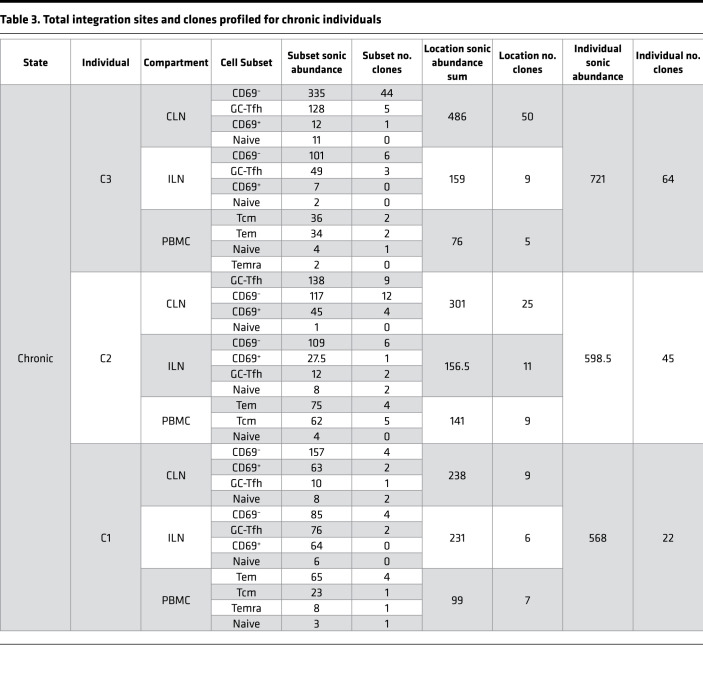
Total integration sites and clones profiled for chronic individuals

**Table 2:**
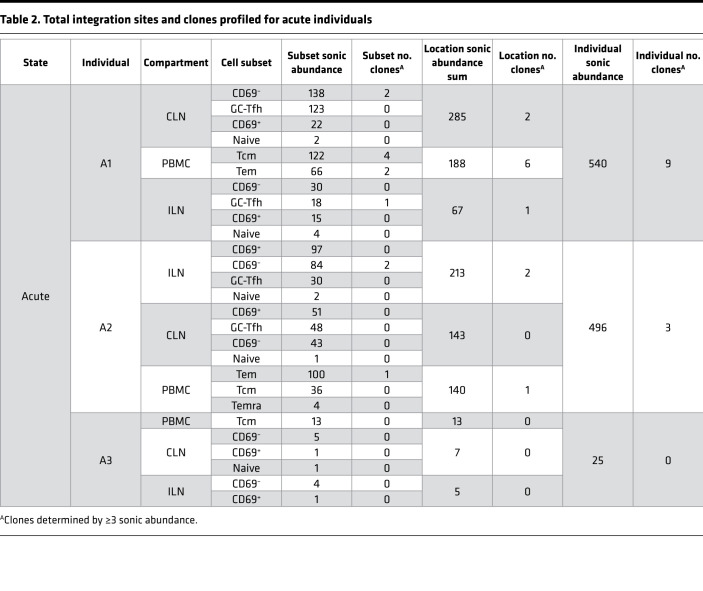
Total integration sites and clones profiled for acute individuals

**Table 1:**
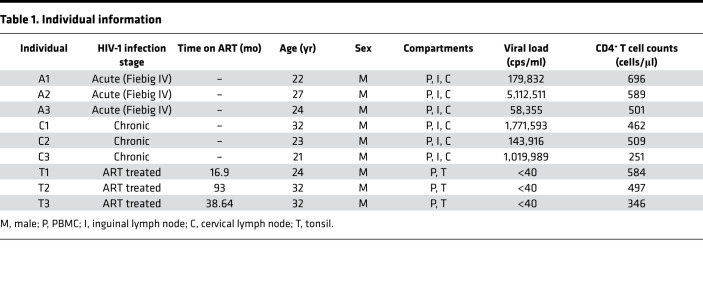
Individual information

## Version 1 09/24/2020

In-Press Preview

## Version 2 10/15/2020

Electronic publication

## Version 3 12/21/2020

Updated author list and contributions
