# Supplementary material for: Assessment of HIV-1 integration in tissues and subsets across infection stages
Source: JCI Insight. 2020 Oct 15;5(20):e139783. doi: 10.1172/jci.insight.139783 (PMC7605534; doi:10.1172/jci.insight.139783)
Supplement: Supplemental data [file jciinsight-5-139783-s011.pdf]

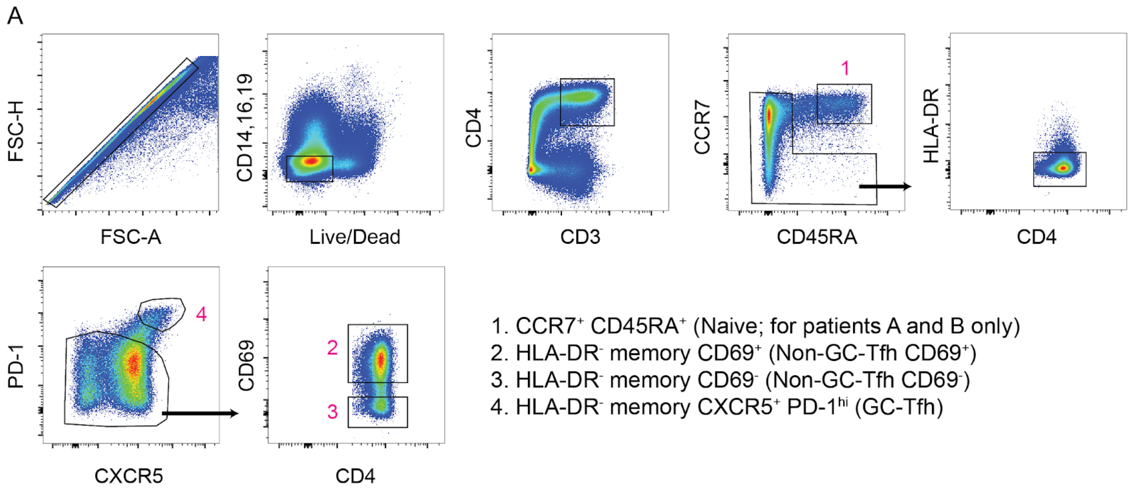

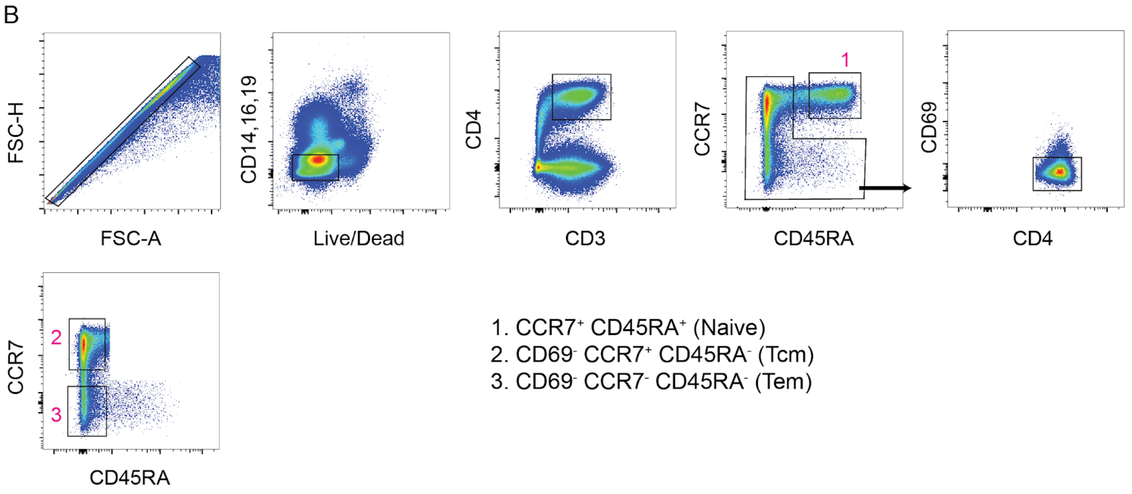

Supplemental Figure 1: Sorting strategy for ART-treated samples. Representative flow plots showing the sort strategy for (A) tonsil samples or (B) peripheral blood samples. Subsets are indicated in pink numbers and detailed in the lists by their specific marker combinations and designation.

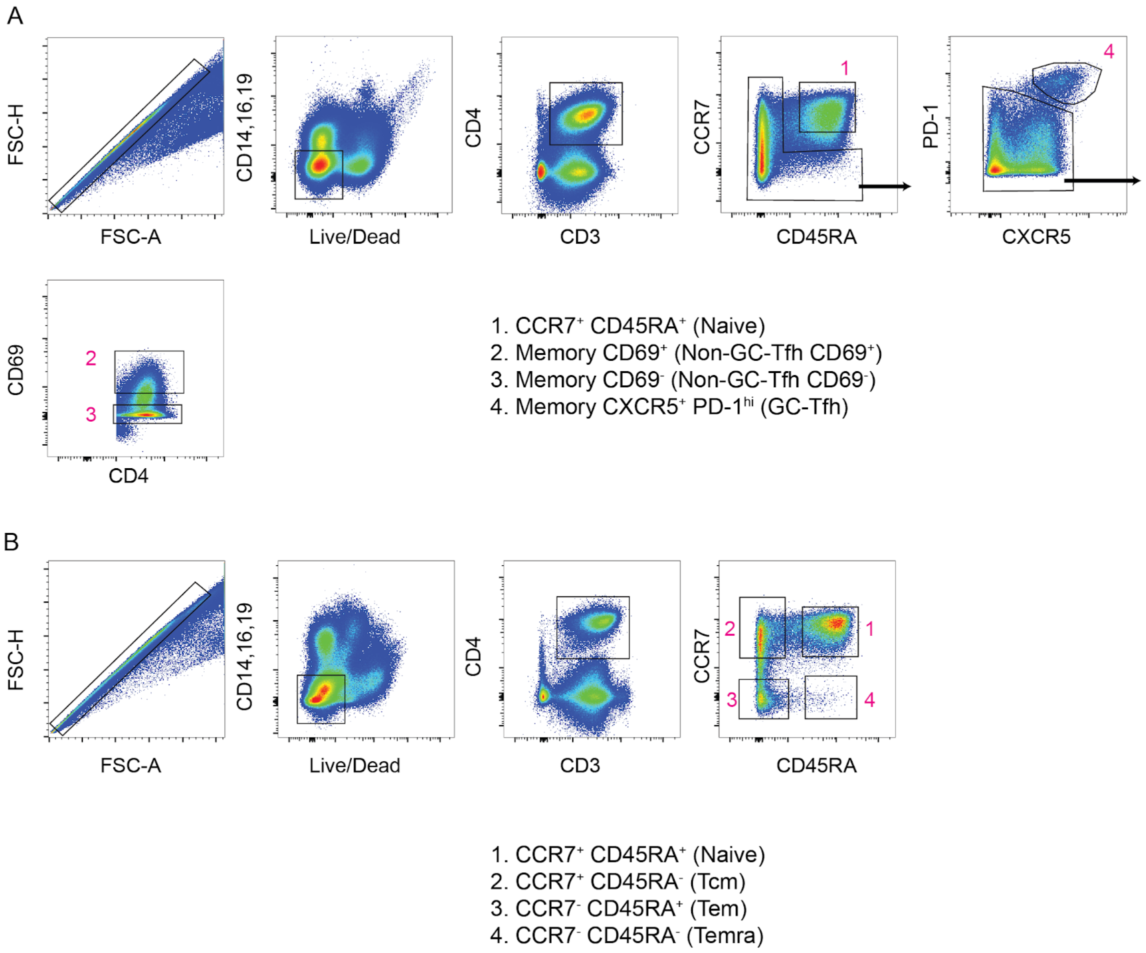

Supplemental Figure 2: Sorting strategy for acute and chronic samples. Representative flow plots showing the sort strategy for (A) lymph node samples or (B) peripheral blood samples. Subsets are indicated in pink numbers and detailed in the lists by their specific marker combinations and designation.

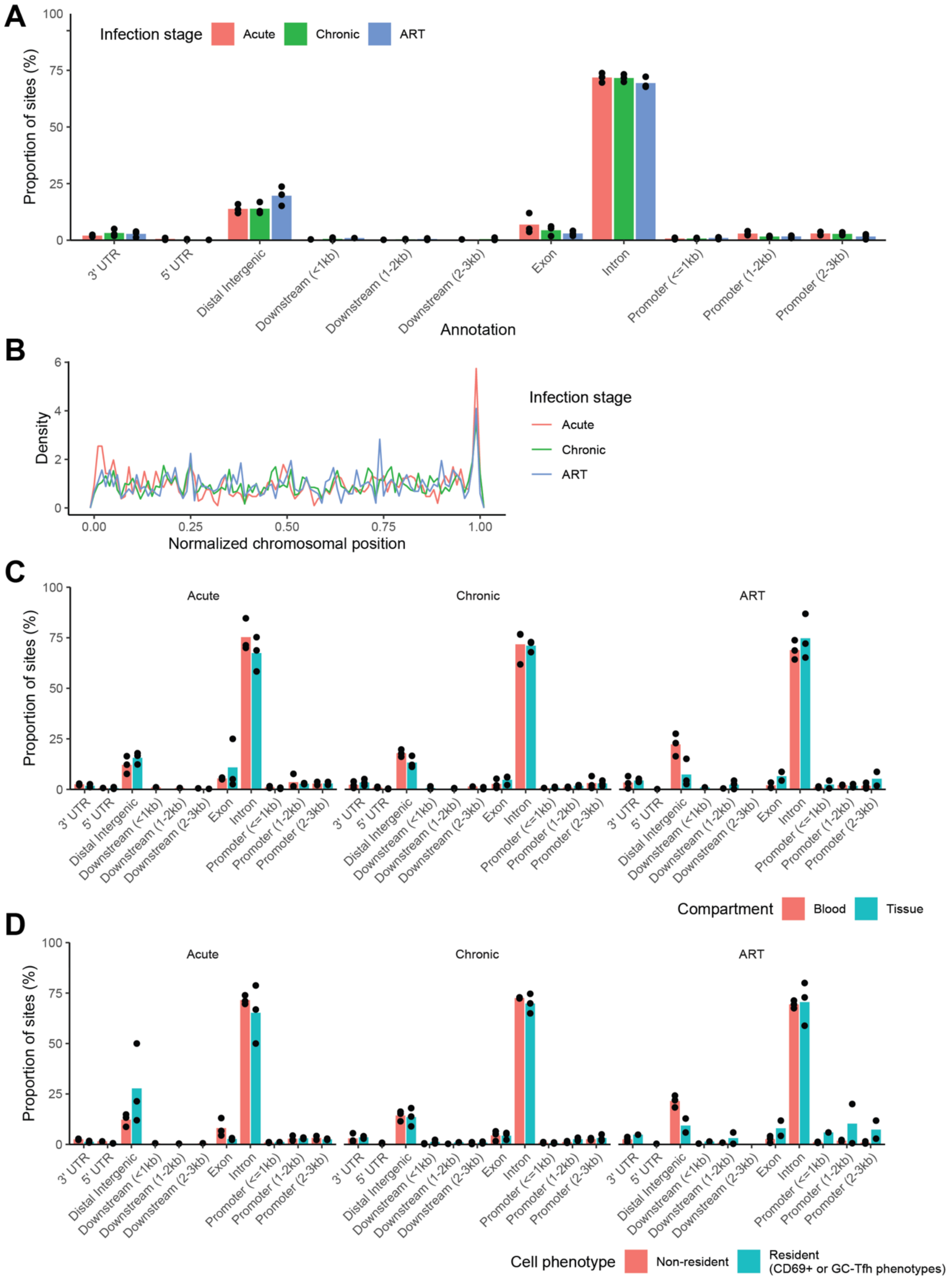

Supplemental Figure 3: Overall characteristics of HIV integration in the entire compiled dataset. (A) Annotation of the sites relative to genic regions is displayed. (B) Sites were

compiled based on their sonic abundance (rounded down to the nearest integer if needed for sites with an estimated non-integer sonic abundance). Normalized chromosomal positions were binned (bin width of 0.01) and a density plot was plotted for sites compiled by infection stage. (C) Annotations relative to genic regions are subsetted based on infection stage and tissue compartment. (D) Annotations relative to genic regions are subsetted based on infection stage and residency subsets. Integration sites were annotated using the ChIPSeeker package in R.

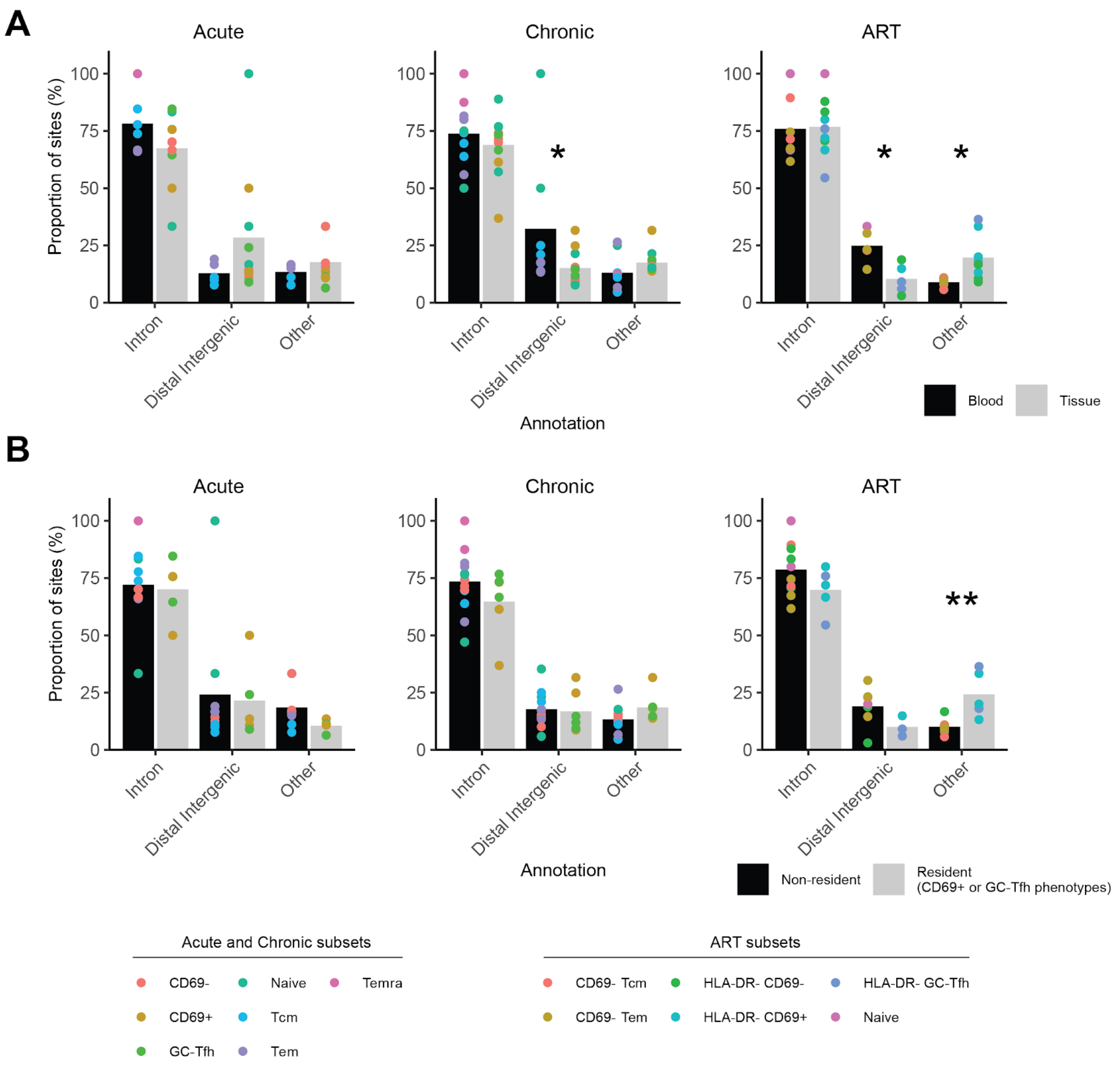

Supplemental Figure 4: Characteristics of HIV integration separated by infection stage, compartment, and residency phenotype. Integration sites were annotated using the ChIPSeeker package in R. Each subset was plotted to assess for differences between intronic, distal intergenic, and other (non-intronic and non-distal intergenic; a full list of these can be seen in Supplemental Figure 3A) annotations. Subsets were grouped by infection stage and (A) tissue compartment or (B) residency phenotype. Statistical comparisons were assessed with Wilcoxon tests. * $p < 0.05$; ** $p < 0.01$

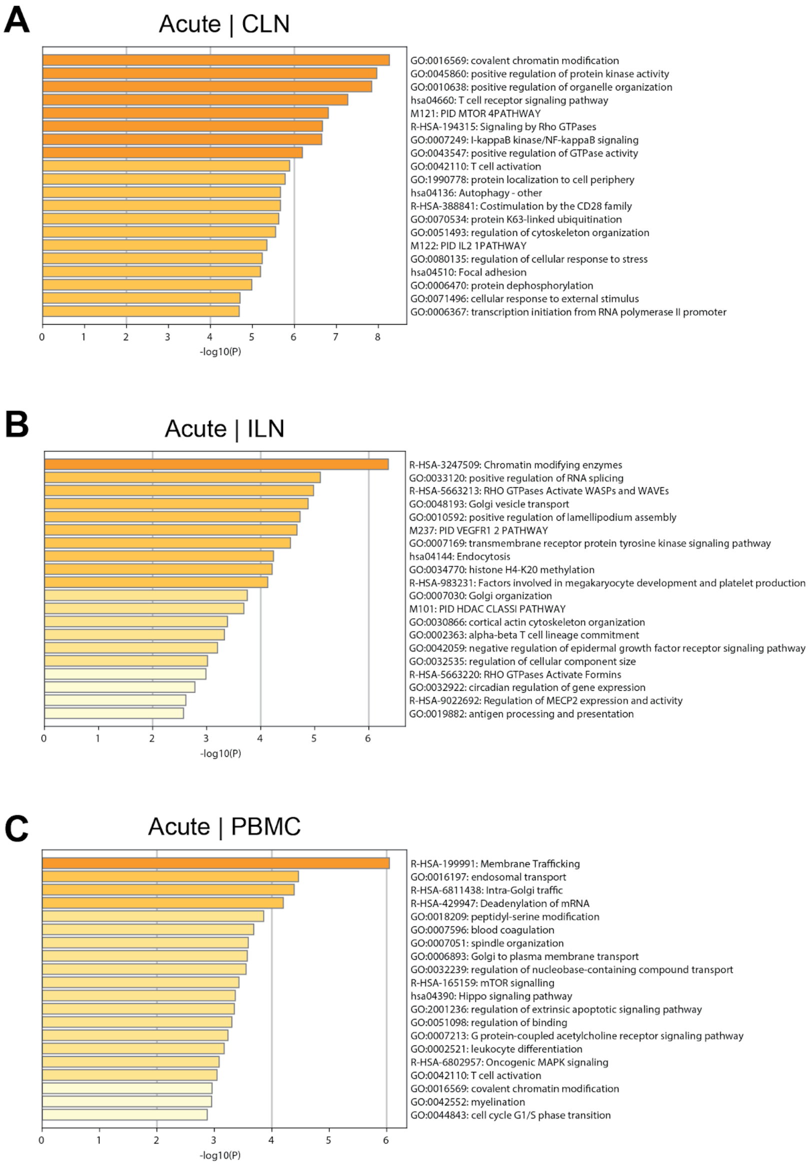

Supplemental Figure 5: Gene ontology analysis for acute samples. Unique integration sites within genic regions were compiled without accounting for abundance. Gene ontology analysis was run on the compiled genes using Metascape for (A) cervical lymph node, (B) inguinal lymph node, and (C) peripheral blood samples.

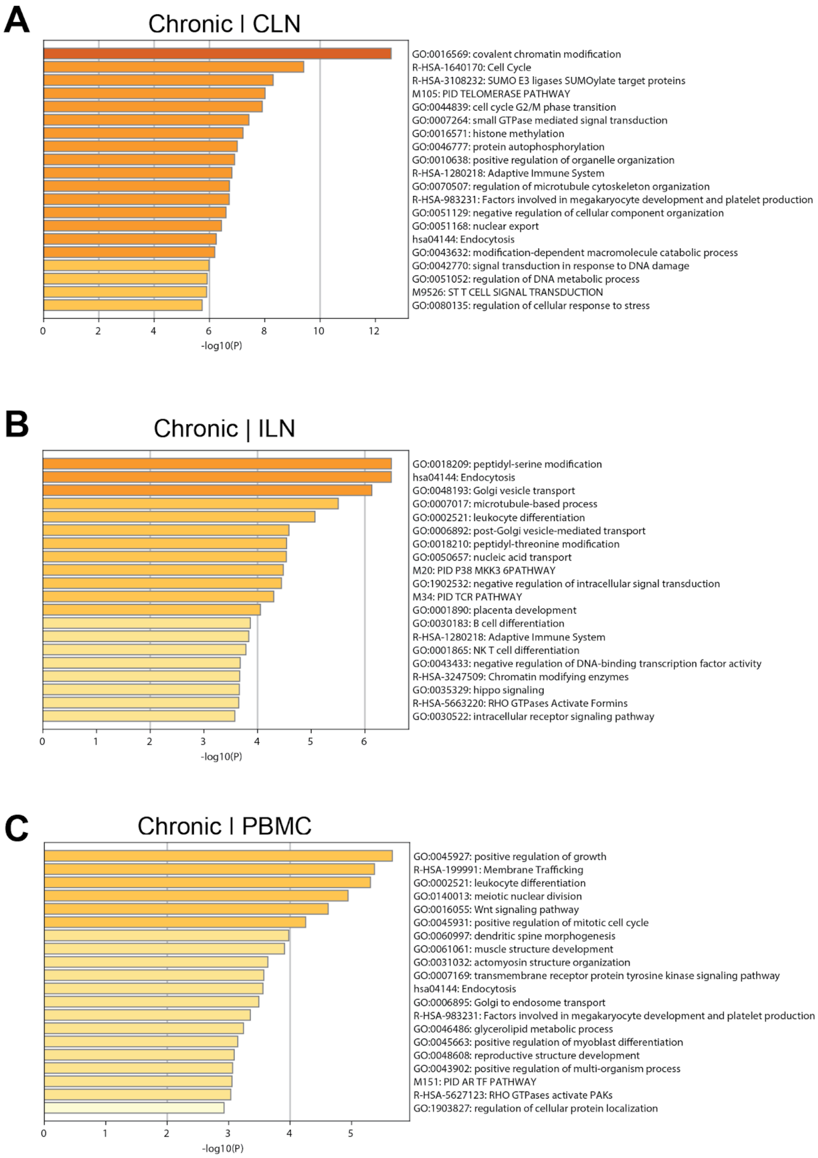

Supplemental Figure 6: Gene ontology analysis for chronic samples. Unique integration sites within genic regions were compiled without accounting for abundance. Gene ontology analysis was run on the compiled genes using Metascape for (A) cervical lymph node, (B) inguinal lymph node, and (C) peripheral blood samples.

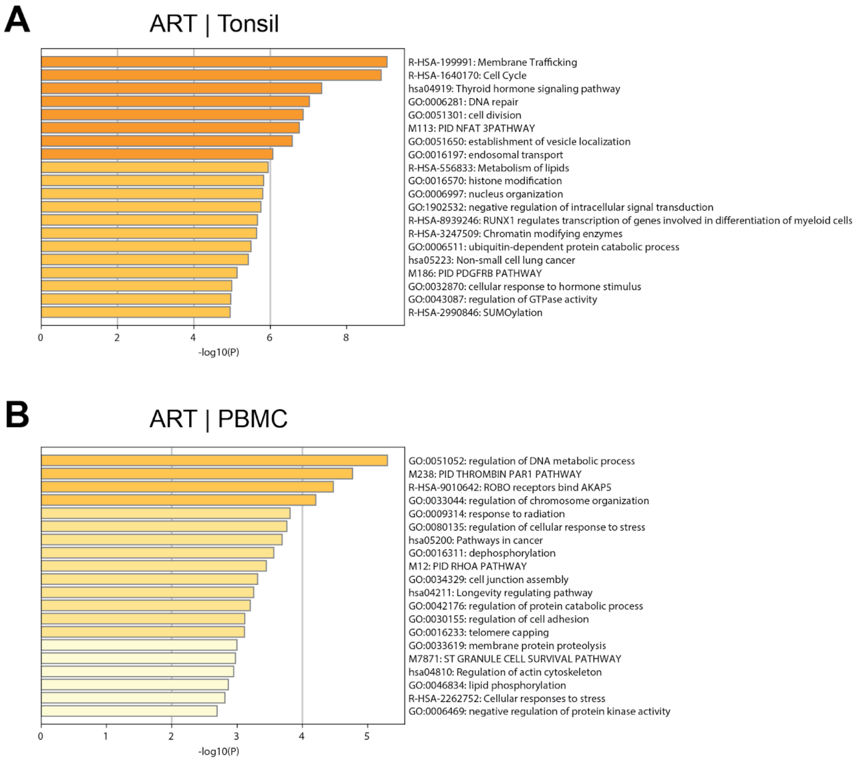

Supplemental Figure 7: Gene ontology analysis for ART-treated samples. Unique integration sites within genic regions were compiled without accounting for abundance. Gene ontology analysis was run on the compiled genes using Metascape for (A) tonsil and (B) PBMC samples.

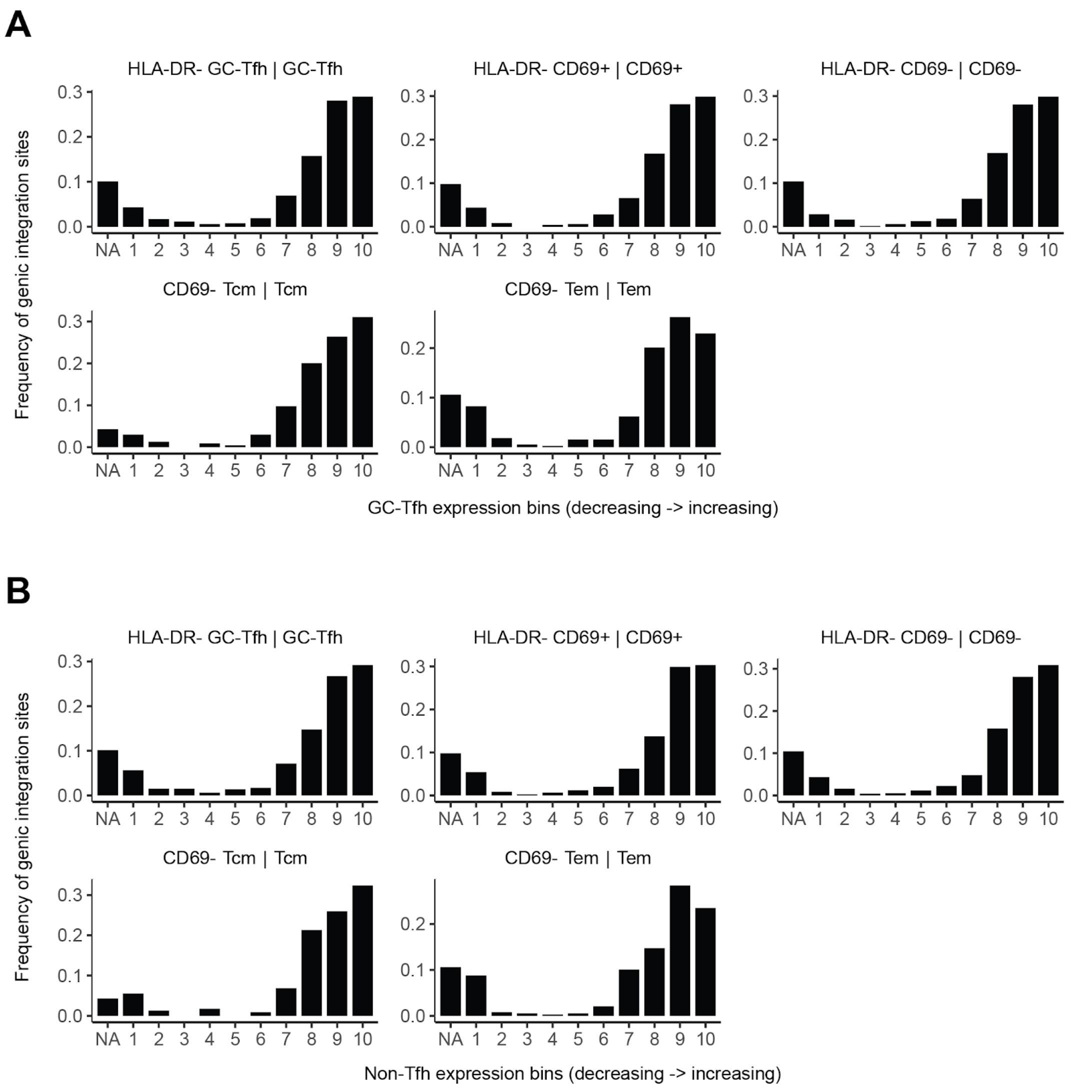

Supplemental Figure 8: Integration sites binned by transcriptional activity. RNAseq datasets on (A) lymph node GC-Tfh and (B) lymph node non-Tfh were binned by increasing levels of transcriptional activity. Genic integration sites grouped by subsets were placed into the appropriate bin and displayed. NA counts genic integration sites where the gene was not detected in the RNAseq experiments.

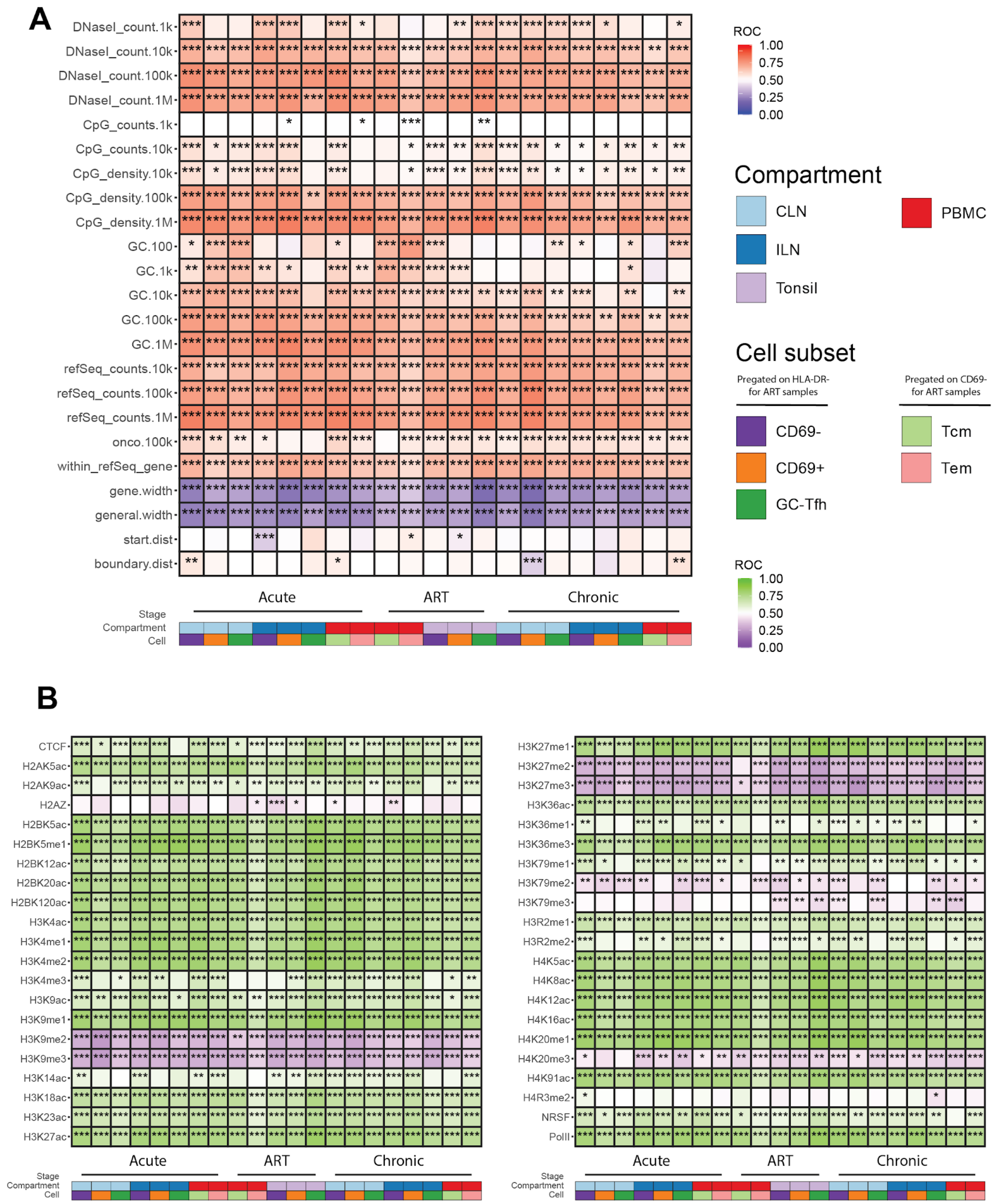

Supplemental Figure 9: Genomic and epigenetic characteristics of HIV integration are comparable across infection stage, compartment, and cell subset. Receiver operating characteristic (ROC) curve analysis was conducted to assess a given annotation's ability to predict actual vs matched random control (MRC) sites. ROC analyses were done for different (A) genomic annotations and (B) epigenetic annotations from previously published studies on

bulk CD4+ T-cells. For genomic annotations, the numeric value at the end of the annotation represents the genome window from the integration site (i.e 10k is 5000 bp ± of integration site). Epigenetic annotations are assessed within a 10kb window. ROC value of 1 indicates that the given annotation was present or observed at a higher level in all actual integration sites over MRC sites. Significance was tested between actual integration sites and MRC sites using a chi-squared test. * $p < 0.05$, ** $p < 0.01$, *** $p < 0.001$

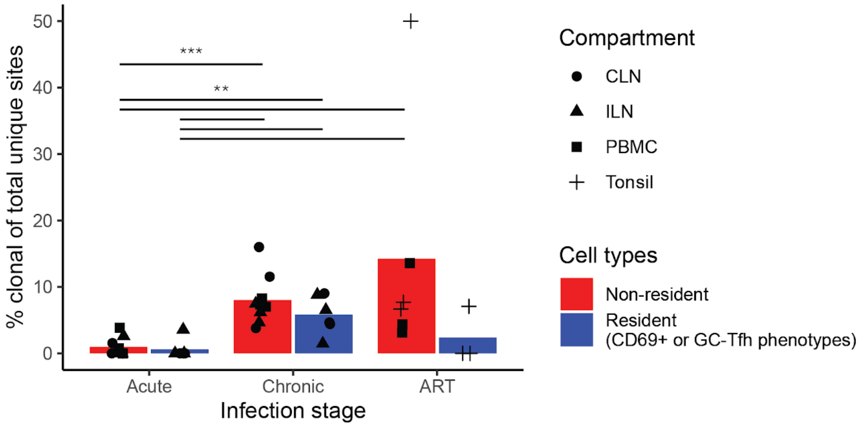

Supplemental Figure 10: Clonal expansion proportions by residency phenotype. Sites were combined across individuals and grouped by residency phenotype. Proportions of clonal sites over total unique sites were then calculated. Bars indicate mean clonal percentage. Significance between different factors was assessed by Wilcoxon Rank Sum test. * $p < 0.5$; ** $p < 0.01$, *** $p < 0.001$.

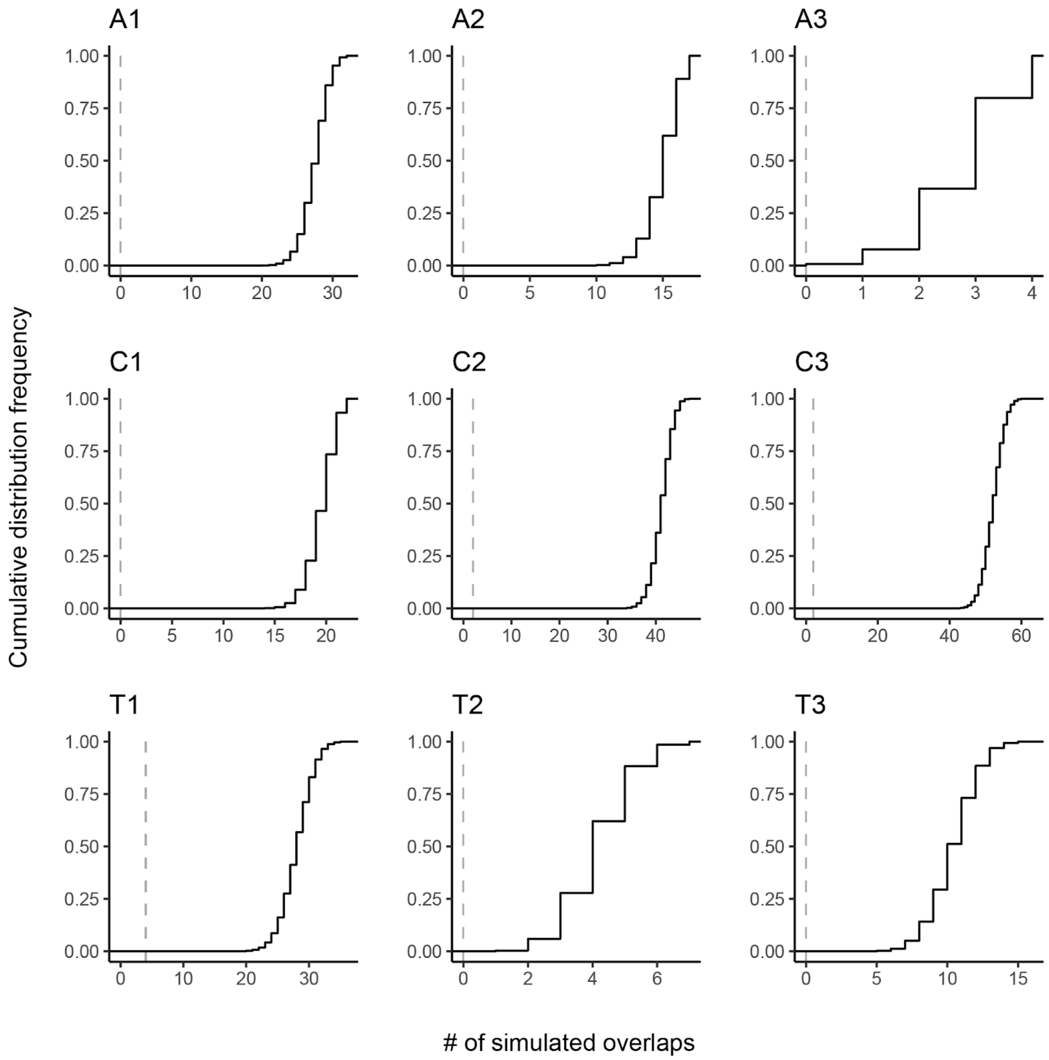

Supplemental Figure 11: Sampling simulation of integration sites by individual. Sites for each individual were compiled while accounting for sonic abundance (abundance was rounded down to the nearest integer if needed). These sites were then randomly sampled into subset/compartment groups based on the original size of the group based on our original sequencing depth. The total number of overlaps across subset/compartment groups were counted. This simulation was run 10,000 times and the number of overlaps is displayed as a cumulative distribution frequency for each individual. The dotted line represents the actual number of overlaps detected in our original dataset.
