# Supplementary material for: Assessment of HIV-1 integration in tissues and subsets across infection stages
Source: JCI Insight. 2020 Oct 15;5(20):e139783. doi: 10.1172/jci.insight.139783 (PMC7605534; doi:10.1172/jci.insight.139783)
Supplement: Supplemental Tables 1-5 [file jciinsight-5-139783-s013.pdf]

Supplemental Table 1: Sort counts for all individuals by subset

Individual	Compartment	Subset	Cells Sorted
A1	CLN	Naive	572035
A1	CLN	CD69+	60933
A1	CLN	CD69-	527767
A1	CLN	GC-Tfh	27146
A1	ILN	Naive	223515
A1	ILN	CD69+	152367
A1	ILN	CD69-	170757
A1	ILN	GC-Tfh	18284
A1	PBMC	Naive	357108
A1	PBMC	Tcm	272419
A1	PBMC	Tem	124110
A1	PBMC	Temra	6943
A2	CLN	Naive	600000
A2	CLN	CD69+	423512
A2	CLN	CD69-	594576
A2	CLN	GC-Tfh	27241
A2	ILN	Naive	600000
A2	ILN	CD69+	600000
A2	ILN	CD69-	600000
A2	ILN	GC-Tfh	31236
A2	PBMC	Naive	368968
A2	PBMC	Tcm	370430
A2	PBMC	Tem	326801
A2	PBMC	Temra	54203
A3	CLN	Naive	298427
A3	CLN	CD69+	119084
A3	CLN	CD69-	206693
A3	CLN	GC-Tfh	4794
A3	ILN	Naive	600000
A3	ILN	CD69+	563028
A3	ILN	CD69-	600000
A3	ILN	GC-Tfh	32990 (est)
A3	PBMC	Naive	301167
A3	PBMC	Tcm	438878
A3	PBMC	Tem	307559
A3	PBMC	Temra	70494
C1	CLN	Naive	292381
C1	CLN	CD69+	427023
C1	CLN	CD69-	118286
C1	CLN	GC-Tfh	10410
C1	ILN	Naive	250000
C1	ILN	CD69+	352598
C1	ILN	CD69-	309110
C1	ILN	GC-Tfh	12946
C1	PBMC	Naive	300000
C1	PBMC	Tcm	328703
C1	PBMC	Tem	99323
C1	PBMC	Temra	9699
C2	CLN	Naive	250000
C2	CLN	CD69+	57482
C2	CLN	CD69-	400000
C2	CLN	GC-Tfh	20470
C2	ILN	Naive	179983
C2	ILN	CD69+	233480
C2	ILN	CD69-	309093
C2	ILN	GC-Tfh	18608
C2	PBMC	Naive	98217
C2	PBMC	Tcm	98051
C2	PBMC	Tem	110528
C2	PBMC	Temra	30732
C3	CLN	Naive	251842
C3	CLN	CD69+	239197
C3	CLN	CD69-	386289
C3	CLN	GC-Tfh	15522
C3	ILN	Naive	149399
C3	ILN	CD69+	76952
C3	ILN	CD69-	169360

C3	ILN	GC-Tfh	2565
C3	PBMC	Naive	160477
C3	PBMC	Tcm	86471
C3	PBMC	Tem	56693
C3	PBMC	Temra	2948
T1	Tonsil	Naive	100951
T1	Tonsil	HLA-DR- CD69+	280330
T1	Tonsil	HLA-DR- CD69-	405538
T1	Tonsil	HLA-DR- GC-Tfh	132190
T1	PBMC	Naive	155961
T1	PBMC	CD69- Tcm	79002
T1	PBMC	CD69- Tem	23208
T2	Tonsil	Naive	26812
T2	Tonsil	HLA-DR- CD69+	196982
T2	Tonsil	HLA-DR- CD69-	229894
T2	Tonsil	HLA-DR- GC-Tfh	34275
T2	PBMC	Naive	175051
T2	PBMC	CD69- Tcm	313144
T2	PBMC	CD69- Tem	109794
T3	Tonsil	HLA-DR- CD69+	277341
T3	Tonsil	HLA-DR- CD69-	192100
T3	Tonsil	HLA-DR- GC-Tfh	200638
T3	PBMC	Naive	132554
T3	PBMC	CD69- Tcm	76688
T3	PBMC	CD69- Tem	180667

Supplemental Table 2: Gene enrichment between acute and ART-treated

Gene*	# Acute sites	# ART sites	# Acute individuals	# ART individuals	Acute Frequency	ART Frequency	% Change
PACS1	5	1	2	1	5.09E-04	1.13E-04	-77.88
PHF21A	3	1	1	1	3.05E-04	1.13E-04	-63.14
RPTOR	3	1	1	1	3.05E-04	1.13E-04	-63.14
NF1	2	1	1	1	2.04E-04	1.13E-04	-44.71
TPD52L2	2	1	2	1	2.04E-04	1.13E-04	-44.71
WASF2	2	1	1	1	2.04E-04	1.13E-04	-44.71
NLRC3	2	1	1	1	2.04E-04	1.13E-04	-44.71
FANCA	2	1	1	1	2.04E-04	1.13E-04	-44.71
CYTH1	2	1	2	1	2.04E-04	1.13E-04	-44.71
SEPT9	4	2	1	1	4.07E-04	2.25E-04	-44.71
DLG1	2	1	2	1	2.04E-04	1.13E-04	-44.71
RHOA	2	1	2	1	2.04E-04	1.13E-04	-44.71
NSD1	2	1	2	1	2.04E-04	1.13E-04	-44.71
STK38	2	1	2	1	2.04E-04	1.13E-04	-44.71
ZNRF2	2	1	2	1	2.04E-04	1.13E-04	-44.71
POLR2A	2	1	1	1	2.04E-04	1.13E-04	-44.71
TBC1D5	2	1	2	1	2.04E-04	1.13E-04	-44.71
ANKRD11	3	2	1	2	3.05E-04	2.25E-04	-26.28
CTDSPL2	1	1	1	1	1.02E-04	1.13E-04	10.59
ARHGDIA	1	1	1	1	1.02E-04	1.13E-04	10.59
HORMAD2	1	1	1	1	1.02E-04	1.13E-04	10.59
PRKAR2A	1	1	1	1	1.02E-04	1.13E-04	10.59
FAM208A	1	1	1	1	1.02E-04	1.13E-04	10.59
CASTOR3	1	1	1	1	1.02E-04	1.13E-04	10.59
FAM126A	1	1	1	1	1.02E-04	1.13E-04	10.59
TBC1D31	1	1	1	1	1.02E-04	1.13E-04	10.59
PPP2R2A	1	1	1	1	1.02E-04	1.13E-04	10.59
TMEM245	1	1	1	1	1.02E-04	1.13E-04	10.59
STX16-NPEPL1	1	1	1	1	1.02E-04	1.12E-04	10.59
LOC100294145	1	1	1	1	1.02E-04	1.12E-04	10.59
MVP	1	1	1	1	1.02E-04	1.13E-04	10.59
CIC	1	1	1	1	1.02E-04	1.13E-04	10.59
CARD8-AS1	1	1	1	1	1.02E-04	1.13E-04	10.59
HNRNPUL2-BSCL2	1	1	1	1	1.02E-04	1.12E-04	10.59
CAP1	1	1	1	1	1.02E-04	1.13E-04	10.59
GLTP	1	1	1	1	1.02E-04	1.13E-04	10.59
CBFB	1	1	1	1	1.02E-04	1.13E-04	10.59
GRB2	1	1	1	1	1.02E-04	1.13E-04	10.59
TLK2	1	1	1	1	1.02E-04	1.13E-04	10.59
OAZ1	1	1	1	1	1.02E-04	1.13E-04	10.59
RELB	1	1	1	1	1.02E-04	1.13E-04	10.59
VAV1	1	1	1	1	1.02E-04	1.13E-04	10.59
DRG1	1	1	1	1	1.02E-04	1.13E-04	10.59
TCTA	1	1	1	1	1.02E-04	1.13E-04	10.59
NSD2	1	1	1	1	1.02E-04	1.13E-04	10.59
COG5	1	1	1	1	1.02E-04	1.13E-04	10.59
PAG1	1	1	1	1	1.02E-04	1.13E-04	10.59
HSF1	1	1	1	1	1.02E-04	1.13E-04	10.59
ELP3	1	1	1	1	1.02E-04	1.13E-04	10.59
HNRNPUL2	1	1	1	1	1.02E-04	1.13E-04	10.59
FGFR1OP2	1	1	1	1	1.02E-04	1.13E-04	10.59
TRAPPC10	1	1	1	1	1.02E-04	1.13E-04	10.59
C6orf106	1	1	1	1	1.02E-04	1.13E-04	10.59
ARID4B	1	1	1	1	1.02E-04	1.13E-04	10.59
SRSF10	1	1	1	1	1.02E-04	1.13E-04	10.59
CAPZA1	1	1	1	1	1.02E-04	1.13E-04	10.59
SETDB1	1	1	1	1	1.02E-04	1.13E-04	10.59
FCHSD2	1	1	1	1	1.02E-04	1.13E-04	10.59
PPP6R3	1	1	1	1	1.02E-04	1.13E-04	10.59
MYCBP2	1	1	1	1	1.02E-04	1.13E-04	10.59
RAB27A	1	1	1	1	1.02E-04	1.13E-04	10.59
ZNF609	1	1	1	1	1.02E-04	1.13E-04	10.59
ATXN2L	1	1	1	1	1.02E-04	1.13E-04	10.59
TANGO6	1	1	1	1	1.02E-04	1.13E-04	10.59
UBE2G1	2	2	1	1	2.04E-04	2.25E-04	10.59
NPLOC4	1	1	1	1	1.02E-04	1.13E-04	10.59
WDR45B	1	1	1	1	1.02E-04	1.13E-04	10.59
STXBP4	1	1	1	1	1.02E-04	1.13E-04	10.59
TTC39C	1	1	1	1	1.02E-04	1.13E-04	10.59
AKAP8L	1	1	1	1	1.02E-04	1.13E-04	10.59
ZNF430	1	1	1	1	1.02E-04	1.13E-04	10.59
LSM14A	1	1	1	1	1.02E-04	1.13E-04	10.59
IFNAR2	1	1	1	1	1.02E-04	1.13E-04	10.59
TNRC6B	1	1	1	1	1.02E-04	1.13E-04	10.59
MARCH3	1	1	1	1	1.02E-04	1.13E-04	10.59
ZFAND3	1	1	1	1	1.02E-04	1.13E-04	10.59
PPP3CC	1	1	1	1	1.02E-04	1.13E-04	10.59

GON4L	1	1	1	1	1.02E-04	1.13E-04	10.59
CDC42	1	1	1	1	1.02E-04	1.13E-04	10.59
MIER1	1	1	1	1	1.02E-04	1.13E-04	10.59
RBM4B	1	1	1	1	1.02E-04	1.13E-04	10.59
KMT5B	1	1	1	1	1.02E-04	1.13E-04	10.59
RNF10	1	1	1	1	1.02E-04	1.13E-04	10.59
PTPN9	1	1	1	2	1.02E-04	1.13E-04	10.59
NPRL3	1	1	1	1	1.02E-04	1.13E-04	10.59
PRKCB	1	1	1	1	1.02E-04	1.13E-04	10.59
ITGAL	1	1	1	1	1.02E-04	1.13E-04	10.59
YWHAE	1	1	1	1	1.02E-04	1.13E-04	10.59
NAA38	1	1	1	1	1.02E-04	1.13E-04	10.59
SBNO2	1	1	1	1	1.02E-04	1.13E-04	10.59
CARD8	1	1	1	1	1.02E-04	1.13E-04	10.59
NOSIP	1	1	1	1	1.02E-04	1.13E-04	10.59
MED16	1	1	1	1	1.02E-04	1.13E-04	10.59
MED25	1	1	1	1	1.02E-04	1.13E-04	10.59
ASXL2	1	1	1	1	1.02E-04	1.13E-04	10.59
STX16	1	1	1	1	1.02E-04	1.13E-04	10.59
PRPF6	1	1	1	1	1.02E-04	1.13E-04	10.59
MRTFA	2	2	2	2	2.04E-04	2.25E-04	10.59
WDR70	1	1	1	1	1.02E-04	1.13E-04	10.59
CCND3	1	1	1	1	1.02E-04	1.13E-04	10.59
MROH1	2	2	1	1	2.04E-04	2.25E-04	10.59
RABL6	1	1	1	1	1.02E-04	1.13E-04	10.59
KDM2A	3	3	2	1	3.05E-04	3.38E-04	10.59
ZGPAT	3	3	2	1	3.05E-04	3.38E-04	10.59
RABEP1	2	3	2	1	2.04E-04	3.38E-04	65.88
BOLA2-SMG1P6	1	2	1	1	1.02E-04	2.25E-04	121.17
LOC606724	1	2	1	1	1.02E-04	2.25E-04	121.17
EYA3	1	2	1	2	1.02E-04	2.25E-04	121.17
TESPA1	1	2	1	1	1.02E-04	2.25E-04	121.17
RBFOX1	1	2	1	2	1.02E-04	2.25E-04	121.17
USP36	1	2	1	1	1.02E-04	2.25E-04	121.17
RNF213	1	2	1	1	1.02E-04	2.25E-04	121.17
ATXN10	1	2	1	1	1.02E-04	2.25E-04	121.17
AP3B1	1	2	1	2	1.02E-04	2.25E-04	121.17
STAG3	1	2	1	1	1.02E-04	2.25E-04	121.17
SMG1P6	1	3	1	1	1.02E-04	3.38E-04	231.76
PBX4	1	3	1	2	1.02E-04	3.38E-04	231.76
FOXK2	1	3	1	2	1.02E-04	3.38E-04	231.76

* Only showing genes that were identified at least once in both groups in any given pairwise comparison

Supplemental Table 3: Gene enrichment between acute and chronic infection

Gene*	# Acute sites	# Chronic sites	# Acute individuals	# Chronic individuals	Acute Frequency	Chronic Frequency	% Change
SEPT9	4	1	1	2	4.07E-04	6.01E-05	-85.23
LINC01578	3	1	1	1	3.05E-04	6.01E-05	-80.3
ABCA7	3	1	2	1	3.05E-04	6.01E-05	-80.3
VPS52	3	1	2	1	3.05E-04	6.01E-05	-80.3
ATL3	2	1	1	1	2.04E-04	6.01E-05	-70.46
PRH1-PRR4	2	1	1	1	2.03E-04	6.01E-05	-70.46
PRH1	2	1	1	1	2.04E-04	6.01E-05	-70.46
PRH1-TAS2R14	2	1	1	1	2.03E-04	6.01E-05	-70.46
GPATCH8	2	1	2	1	2.04E-04	6.01E-05	-70.46
TPD52L2	2	1	2	1	2.04E-04	6.01E-05	-70.46
NSD1	2	1	2	1	2.04E-04	6.01E-05	-70.46
NVL	2	1	2	1	2.04E-04	6.02E-05	-70.46
VPS45	2	1	2	1	2.04E-04	6.01E-05	-70.46
FANCA	2	1	1	1	2.04E-04	6.01E-05	-70.46
CYTH1	2	1	2	1	2.04E-04	6.01E-05	-70.46
POLR2A	2	1	1	1	2.04E-04	6.01E-05	-70.46
CHD2	3	2	2	2	3.05E-04	1.2E-04	-60.61
ZZEF1	3	2	1	2	3.05E-04	1.2E-04	-60.61
RPTOR	3	2	1	2	3.05E-04	1.2E-04	-60.61
USP34	3	2	1	3	3.05E-04	1.2E-04	-60.61
ANKRD11	3	2	1	1	3.05E-04	1.2E-04	-60.61
PACS1	5	4	2	3	5.09E-04	2.41E-04	-52.73
LINC01260	1	1	1	1	1.02E-04	6.01E-05	-40.91
LINC-PINT	1	1	1	1	1.02E-04	6.01E-05	-40.91
STXBP3	1	1	1	1	1.02E-04	6.01E-05	-40.91
FLVCR1	1	1	1	1	1.02E-04	6.01E-05	-40.91
ARL 3.00	2	2	2	2	2.04E-04	1.2E-04	-40.91
SIK3	1	1	1	1	1.02E-04	6.01E-05	-40.91
FCHSD2	1	1	1	1	1.02E-04	6.01E-05	-40.91
MALAT1	1	1	1	1	1.02E-04	6.01E-05	-40.91
GRK2	1	1	1	1	1.02E-04	6.01E-05	-40.91
SCAF11	1	1	1	1	1.02E-04	6.01E-05	-40.91
ZNF384	1	1	1	1	1.02E-04	6.01E-05	-40.91
ARID4A	1	1	1	1	1.02E-04	6.01E-05	-40.91
ZNF609	1	1	1	1	1.02E-04	6.01E-05	-40.91
AKAP13	1	1	1	1	1.02E-04	6.01E-05	-40.91
IQGAP1	1	1	1	1	1.02E-04	6.01E-05	-40.91
SMG1P6	1	1	1	2	1.02E-04	6.01E-05	-40.91
BOLA2-SMG1P6	1	1	1	2	1.02E-04	6.01E-05	-40.91
JPT2	1	1	1	1	1.02E-04	6.01E-05	-40.91
ATXN2L	1	1	1	1	1.02E-04	6.01E-05	-40.91
ZNF267	1	1	1	1	1.02E-04	6.01E-05	-40.91
CBFB	1	1	1	1	1.02E-04	6.01E-05	-40.91
TANGO6	1	1	1	1	1.02E-04	6.01E-05	-40.91
WWP2	1	1	1	1	1.02E-04	6.01E-05	-40.91
UBE2G1	2	2	1	2	2.04E-04	1.2E-04	-40.91
SKA2	1	1	1	1	1.02E-04	6.01E-05	-40.91
MIR454	1	1	1	1	1.02E-04	6.01E-05	-40.91
WDR45B	1	1	1	1	1.02E-04	6.01E-05	-40.91
SUPT6H	1	1	1	1	1.02E-04	6.01E-05	-40.91
RABEP1	2	2	2	2	2.04E-04	1.2E-04	-40.91
TTC39C	1	1	1	1	1.02E-04	6.01E-05	-40.91
HSPBP1	1	1	1	1	1.02E-04	6.01E-05	-40.91
CNN2	1	1	1	1	1.02E-04	6.01E-05	-40.91
DNM2	1	1	1	1	1.02E-04	6.01E-05	-40.91
TPM4	1	1	1	1	1.02E-04	6.01E-05	-40.91
LSM14A	1	1	1	1	1.02E-04	6.01E-05	-40.91
ZNF383	1	1	1	1	1.02E-04	6.01E-05	-40.91
HDGFL2	1	1	1	1	1.02E-04	6.01E-05	-40.91
CLPTM1	1	1	1	1	1.02E-04	6.01E-05	-40.91
EMP3	1	1	1	2	1.02E-04	6.01E-05	-40.91
ZNF460	1	1	1	1	1.02E-04	6.01E-05	-40.91
HNRNPM	1	1	1	1	1.02E-04	6.01E-05	-40.91
ARID3A	1	1	1	1	1.02E-04	6.01E-05	-40.91
SOS 1	1	1	1	1	1.02E-04	6.01E-05	-40.91
STARD7	1	1	1	1	1.02E-04	6.01E-05	-40.91
MAP4K4	1	1	1	1	1.02E-04	6.01E-05	-40.91
GIGYF2	1	1	1	1	1.02E-04	6.01E-05	-40.91
STARD7-AS1	1	1	1	1	1.02E-04	6.01E-05	-40.91
IL10RB	1	1	1	1	1.02E-04	6.01E-05	-40.91
ADARB1	1	1	1	1	1.02E-04	6.01E-05	-40.91
DEPDC5	1	1	1	1	1.02E-04	6.01E-05	-40.91
RHOA	2	2	2	2	2.04E-04	1.2E-04	-40.91
ATP1B3	1	1	1	1	1.02E-04	6.01E-05	-40.91
VTA1	1	1	1	1	1.02E-04	6.01E-05	-40.91

UTRN	1	1	1	1	1.02E-04	6.01E-05	-40.91
AHR	1	1	1	1	1.02E-04	6.02E-05	-40.91
BOP 1.00	1	1	1	1	1.02E-04	6.01E-05	-40.91
PAG1	1	1	1	1	1.02E-04	6.01E-05	-40.91
HSF1	1	1	1	1	1.02E-04	6.01E-05	-40.91
RPS6	1	1	1	1	1.02E-04	6.01E-05	-40.91
MAN1B1	1	1	1	1	1.02E-04	6.01E-05	-40.91
DOCK11	1	1	1	1	1.02E-04	6.01E-05	-40.91
CDC42	1	1	1	1	1.02E-04	6.01E-05	-40.91
KIF5B	1	1	1	1	1.02E-04	6.01E-05	-40.91
PARD3	1	1	1	1	1.02E-04	6.01E-05	-40.91
SPAG6	1	1	1	1	1.02E-04	6.01E-05	-40.91
APBB1IP	1	1	1	1	1.02E-04	6.01E-05	-40.91
NUP98	1	1	1	1	1.02E-04	6.01E-05	-40.91
TMEM116	1	1	1	2	1.02E-04	6.01E-05	-40.91
RNF10	1	1	1	1	1.02E-04	6.01E-05	-40.91
USP15	1	1	1	1	1.02E-04	6.01E-05	-40.91
DOCK9	1	1	1	1	1.02E-04	6.01E-05	-40.91
PSMB5	1	1	1	1	1.02E-04	6.01E-05	-40.91
DENND4A	1	1	1	1	1.02E-04	6.01E-05	-40.91
PTPN9	1	1	1	1	1.02E-04	6.01E-05	-40.91
MOSMO	2	2	1	1	2.04E-04	1.2E-04	-40.91
FAM222B	1	1	1	1	1.02E-04	6.01E-05	-40.91
FOXK2	1	1	1	1	1.02E-04	6.01E-05	-40.91
SBNO2	1	1	1	1	1.02E-04	6.01E-05	-40.91
MOB3A	1	1	1	1	1.02E-04	6.01E-05	-40.91
KCNN4	1	1	1	1	1.02E-04	6.01E-05	-40.91
CARD8	1	1	1	1	1.02E-04	6.01E-05	-40.91
GATAD2A	1	1	1	1	1.02E-04	6.01E-05	-40.91
SIPA1L3	1	1	1	1	1.02E-04	6.01E-05	-40.91
SPATS2L	1	1	1	1	1.02E-04	6.01E-05	-40.91
NCOA6	1	1	1	1	1.02E-04	6.01E-05	-40.91
ZNFX1	1	1	1	1	1.02E-04	6.01E-05	-40.91
TSHZ2	1	1	1	1	1.02E-04	6.01E-05	-40.91
MRTFA	2	2	2	2	2.04E-04	1.2E-04	-40.91
TCF20	1	1	1	1	1.02E-04	6.01E-05	-40.91
EIF3L	1	1	1	1	1.02E-04	6.01E-05	-40.91
STAG1	1	1	1	1	1.02E-04	6.01E-05	-40.91
SETD2	1	1	1	1	1.02E-04	6.01E-05	-40.91
IP6K2	1	1	1	1	1.02E-04	6.01E-05	-40.91
PRKAR2A	1	1	1	1	1.02E-04	6.01E-05	-40.91
PFDN1	1	1	1	1	1.02E-04	6.01E-05	-40.91
AP3B1	1	1	1	1	1.02E-04	6.01E-05	-40.91
PARP8	1	1	1	1	1.02E-04	6.01E-05	-40.91
HCG25	1	1	1	1	1.02E-04	6.01E-05	-40.91
STAG3	1	1	1	1	1.02E-04	6.01E-05	-40.91
AGFG2	2	2	1	2	2.04E-04	1.2E-04	-40.91
TAX1BP1	1	1	1	1	1.02E-04	6.01E-05	-40.91
ZNF34	1	1	1	2	1.02E-04	6.01E-05	-40.91
ARFGEF1	1	1	1	1	1.02E-04	6.01E-05	-40.91
CAMSAP1	1	1	1	2	1.02E-04	6.01E-05	-40.91
FAM120A	1	1	1	1	1.02E-04	6.01E-05	-40.91
KDM6A	1	1	1	1	1.02E-04	6.01E-05	-40.91
CD27-AS1	1	1	1	1	1.02E-04	6.01E-05	-40.91
CEACAM21	2	2	1	1	2.04E-04	1.2E-04	-40.91
KDM2A	3	4	2	2	3.05E-04	2.41E-04	-21.22
VMP1	2	3	2	2	2.04E-04	1.8E-04	-11.37
MROH1	2	3	1	3	2.04E-04	1.8E-04	-11.37
GNB1	1	2	1	2	1.02E-04	1.2E-04	18.17
SPEN	1	2	1	2	1.02E-04	1.2E-04	18.17
EHD1	1	2	1	1	1.02E-04	1.2E-04	18.17
CTCF	1	2	1	1	1.02E-04	1.2E-04	18.17
GRB2	1	2	1	2	1.02E-04	1.2E-04	18.17
TCF3	1	2	1	2	1.02E-04	1.2E-04	18.17
VAV1	1	2	1	2	1.02E-04	1.2E-04	18.17
STK4	1	2	1	1	1.02E-04	1.2E-04	18.17
UBR5	1	2	1	2	1.02E-04	1.2E-04	18.17
ARID4B	1	2	1	2	1.02E-04	1.2E-04	18.17
UBAP2L	1	2	1	2	1.02E-04	1.2E-04	18.17
PPP6R3	1	2	1	3	1.02E-04	1.2E-04	18.17
TNRC6B	1	2	1	2	1.02E-04	1.2E-04	18.17
RPS6KB1	1	2	1	1	1.02E-04	1.2E-04	18.17
HORMAD2	1	2	1	2	1.02E-04	1.2E-04	18.17
FAM126A	1	2	1	2	1.02E-04	1.2E-04	18.17
ARHGAP45	1	2	1	2	1.02E-04	1.2E-04	18.17
HNRNPUL1	1	2	1	2	1.02E-04	1.2E-04	18.17
C6orf106	1	2	1	2	1.02E-04	1.2E-04	18.17
GON4L	1	2	1	1	1.02E-04	1.2E-04	18.17
KMT2A	1	2	1	2	1.02E-04	1.2E-04	18.17
NLRC5	1	2	1	2	1.02E-04	1.2E-04	18.17
IKZF3	1	2	1	1	1.02E-04	1.2E-04	18.17

SKAP1	1	2	1	2	1.02E-04	1.2E-04	18.17
TAOK1	1	2	1	2	1.02E-04	1.2E-04	18.17
STK11	1	2	1	2	1.02E-04	1.2E-04	18.17
ODF3B	1	2	1	2	1.02E-04	1.2E-04	18.17
SPON2	1	2	1	1	1.02E-04	1.2E-04	18.17
ZC3H3	1	2	1	3	1.02E-04	1.2E-04	18.17
LRBA	2	5	1	2	2.04E-04	3.01E-04	47.71
MYCBP2	1	3	1	2	1.02E-04	1.8E-04	77.26
MVP	1	3	1	2	1.02E-04	1.8E-04	77.26
NPLOC4	1	3	1	2	1.02E-04	1.8E-04	77.26
MAP3K14	1	3	1	3	1.02E-04	1.8E-04	77.26
SARNP	1	3	1	3	1.02E-04	1.8E-04	77.26
YWHAE	1	3	1	2	1.02E-04	1.8E-04	77.26
BRIP1	1	3	1	2	1.02E-04	1.8E-04	77.26
NOSIP	1	3	1	2	1.02E-04	1.8E-04	77.26
TRAPPC10	1	3	1	3	1.02E-04	1.8E-04	77.26
IP6K1	1	3	1	2	1.02E-04	1.8E-04	77.26
LOC100294145	1	4	1	3	1.02E-04	2.4E-04	136.34

* Only showing genes that were identified at least once in both groups in any given pairwise comparison

Supplemental Table 4: Gene enrichment between chronic and ART-treated

Gene*	# Chronic sites	# ART sites	# Chronic individuals	# ART individuals	Chronic Frequency	ART Frequency	% Change
LOC100294145	4	1	3	1	2.4E-04	1.12E-04	-53.21
PACS1	4	1	3	1	2.41E-04	1.13E-04	-53.21
YWHAE	3	1	2	1	1.8E-04	1.13E-04	-37.61
PAFAH1B1	3	1	2	1	1.8E-04	1.13E-04	-37.61
NOSIP	3	1	2	1	1.8E-04	1.13E-04	-37.61
TRAPPC10	3	1	3	1	1.8E-04	1.13E-04	-37.61
GBF1	3	1	2	1	1.8E-04	1.13E-04	-37.61
MVP	3	1	2	1	1.8E-04	1.13E-04	-37.61
MYCBP2	3	1	2	1	1.8E-04	1.13E-04	-37.61
NPLOC4	3	1	2	1	1.8E-04	1.13E-04	-37.61
DNAJC5	3	1	2	1	1.8E-04	1.13E-04	-37.61
PPP2R5C	2	1	2	1	1.2E-04	1.13E-04	-6.42
HORMAD2	2	1	2	1	1.2E-04	1.13E-04	-6.42
FAM126A	2	1	2	1	1.2E-04	1.13E-04	-6.42
RUNX1T1	2	1	1	1	1.2E-04	1.13E-04	-6.42
MIR941-1	2	1	1	1	1.2E-04	1.13E-04	-6.42
MIR941-2	2	1	1	1	1.2E-04	1.13E-04	-6.42
MIR941-3	2	1	1	1	1.2E-04	1.13E-04	-6.42
MIR941-4	2	1	1	1	1.2E-04	1.13E-04	-6.42
MIR941-5	2	1	1	1	1.2E-04	1.13E-04	-6.42
C6orf106	2	1	2	1	1.2E-04	1.13E-04	-6.42
GON4L	2	1	1	1	1.2E-04	1.13E-04	-6.42
MACF1	2	1	1	1	1.2E-04	1.13E-04	-6.42
RPTOR	2	1	2	1	1.2E-04	1.13E-04	-6.42
PHF14	2	1	2	1	1.2E-04	1.13E-04	-6.42
SMG6	2	1	2	1	1.2E-04	1.13E-04	-6.42
GRB2	2	1	2	1	1.2E-04	1.13E-04	-6.42
VAV1	2	1	2	1	1.2E-04	1.13E-04	-6.42
RHOA	2	1	2	1	1.2E-04	1.13E-04	-6.42
OGDH	2	1	2	1	1.2E-04	1.13E-04	-6.42
ARID4B	2	1	2	1	1.2E-04	1.13E-04	-6.42
PPP6R3	2	1	3	1	1.2E-04	1.13E-04	-6.42
TNRC6B	2	1	2	1	1.2E-04	1.13E-04	-6.42
INPP4B	2	1	3	1	1.2E-04	1.13E-04	-6.42
VPS13B	2	1	2	1	1.2E-04	1.13E-04	-6.42
ASH1L	3	2	3	1	1.8E-04	2.25E-04	24.77
MROH1	3	2	3	1	1.8E-04	2.25E-04	24.77
SMG1	3	2	3	2	1.8E-04	2.25E-04	24.77
KDM2A	4	3	2	1	2.41E-04	3.38E-04	40.37
ST3GAL3	1	1	1	1	6.01E-05	1.13E-04	87.16
SUPT16H	1	1	1	1	6.01E-05	1.13E-04	87.16
ANKRD11	2	2	1	2	1.2E-04	2.25E-04	87.16
PIP4K2B	1	1	1	1	6.01E-05	1.13E-04	87.16
SLC44A2	1	1	1	1	6.01E-05	1.13E-04	87.16
TPD52L2	1	1	1	1	6.01E-05	1.13E-04	87.16
POLDIP3	1	1	1	1	6.01E-05	1.13E-04	87.16
SMARCC1	1	1	1	1	6.01E-05	1.13E-04	87.16
PRKAR2A	1	1	1	1	6.01E-05	1.13E-04	87.16
USP24	1	1	1	1	6.01E-05	1.13E-04	87.16
LRIG2	1	1	2	1	6.01E-05	1.13E-04	87.16
CDC42	1	1	1	1	6.01E-05	1.13E-04	87.16
ASCC1	1	1	1	1	6.01E-05	1.13E-04	87.16
SHOC2	1	1	1	1	6.01E-05	1.13E-04	87.16
ARHGEF12	1	1	1	1	6.01E-05	1.13E-04	87.16
C11orf80	1	1	1	1	6.01E-05	1.13E-04	87.16
RNF10	1	1	1	1	6.01E-05	1.13E-04	87.16
MIS18BP1	1	1	1	1	6.01E-05	1.13E-04	87.16
DDX24	1	1	1	1	6.01E-05	1.13E-04	87.16
MARK3	1	1	2	1	6.01E-05	1.13E-04	87.16
KIAA0586	1	1	1	1	6.01E-05	1.13E-04	87.16
PTPN9	1	1	1	2	6.01E-05	1.13E-04	87.16
PIAS1	1	1	2	1	6.01E-05	1.13E-04	87.16
LUC7L	1	1	1	1	6.01E-05	1.13E-04	87.16
FANCA	1	1	1	1	6.01E-05	1.13E-04	87.16
CWC25	1	1	1	1	6.01E-05	1.13E-04	87.16
CYTH1	1	1	1	1	6.01E-05	1.13E-04	87.16
RELCH	1	1	1	1	6.01E-05	1.13E-04	87.16
SBNO2	1	1	1	1	6.01E-05	1.13E-04	87.16
CEP89	1	1	1	1	6.01E-05	1.13E-04	87.16
CARD8	1	1	1	1	6.01E-05	1.13E-04	87.16
ARHGEF18	1	1	1	1	6.01E-05	1.13E-04	87.16
SUMO1	1	1	1	1	6.01E-05	1.13E-04	87.16
ANKRD36C	1	1	1	1	6.01E-05	1.13E-04	87.16
BRWD1	1	1	1	1	6.01E-05	1.13E-04	87.16
DGCR2	1	1	1	1	6.01E-05	1.13E-04	87.16
TTC28	1	1	2	1	6.01E-05	1.13E-04	87.16
MRTFA	2	2	2	2	1.2E-04	2.25E-04	87.16

TRABD	2	2	2	1	1.2E-04	2.25E-04	87.16
RAB7A	1	1	1	1	6.01E-05	1.13E-04	87.16
SETD5	1	1	1	1	6.01E-05	1.13E-04	87.16
CAMK4	1	1	1	1	6.01E-05	1.13E-04	87.16
RCAN2	1	1	1	1	6.01E-05	1.13E-04	87.16
LOC101926915	1	1	1	1	6.01E-05	1.12E-04	87.16
JAZF1	1	1	1	1	6.01E-05	1.13E-04	87.16
ASAP1	1	1	1	1	6.01E-05	1.13E-04	87.16
NBN	1	1	1	1	6.02E-05	1.13E-04	87.16
ASTN2	1	1	1	1	6.01E-05	1.13E-04	87.16
UBAP2	1	1	1	1	6.01E-05	1.13E-04	87.16
MIR1268A	2	2	1	1	1.2E-04	2.25E-04	87.16
CS	1	1	1	1	6.02E-05	1.13E-04	87.16
TRIM33	1	1	1	1	6.01E-05	1.13E-04	87.16
FAF1	1	1	1	1	6.01E-05	1.13E-04	87.16
ATE1	1	1	1	1	6.01E-05	1.13E-04	87.16
EPC1	1	1	1	1	6.01E-05	1.13E-04	87.16
PPP3CB	1	1	1	1	6.01E-05	1.13E-04	87.16
WAPL	1	1	1	1	6.01E-05	1.13E-04	87.16
FCHSD2	1	1	1	1	6.01E-05	1.13E-04	87.16
FAR1	1	1	1	1	6.01E-05	1.13E-04	87.16
DPF2	1	1	1	1	6.01E-05	1.13E-04	87.16
ZCCHC8	1	1	1	1	6.01E-05	1.13E-04	87.16
PYM1	1	1	1	1	6.01E-05	1.13E-04	87.16
SETD1B	1	1	1	1	6.01E-05	1.13E-04	87.16
WNK1	1	1	1	1	6.01E-05	1.13E-04	87.16
ERC1	1	1	1	1	6.01E-05	1.13E-04	87.16
TPP2	1	1	1	1	6.01E-05	1.13E-04	87.16
ATP8A2	1	1	1	1	6.01E-05	1.13E-04	87.16
CIPC	1	1	1	1	6.01E-05	1.13E-04	87.16
UBR1	1	1	1	1	6.01E-05	1.13E-04	87.16
ZNF609	1	1	1	1	6.01E-05	1.13E-04	87.16
UBE2Q2	1	1	2	1	6.01E-05	1.13E-04	87.16
CREBBP	2	2	1	1	1.2E-04	2.25E-04	87.16
ATXN2L	1	1	1	1	6.01E-05	1.13E-04	87.16
CBFB	1	1	1	1	6.01E-05	1.13E-04	87.16
NFATC3	1	1	1	1	6.01E-05	1.13E-04	87.16
TANGO6	1	1	1	1	6.01E-05	1.13E-04	87.16
SSH2	1	1	1	1	6.01E-05	1.13E-04	87.16
UBE2G1	2	2	2	1	1.2E-04	2.25E-04	87.16
SMURF2	1	1	1	1	6.01E-05	1.13E-04	87.16
WDR45B	1	1	1	1	6.01E-05	1.13E-04	87.16
HEXIM2	1	1	1	1	6.01E-05	1.13E-04	87.16
CLTC	1	1	2	1	6.01E-05	1.13E-04	87.16
POLR2A	1	1	1	1	6.01E-05	1.13E-04	87.16
MFSD11	1	1	2	1	6.01E-05	1.13E-04	87.16
TTC39C	1	1	1	1	6.01E-05	1.13E-04	87.16
ZNF555	1	1	1	1	6.01E-05	1.13E-04	87.16
LSM14A	1	1	1	1	6.01E-05	1.13E-04	87.16
TTC21B	1	1	1	1	6.01E-05	1.13E-04	87.16
CUL3	1	1	1	1	6.01E-05	1.13E-04	87.16
ATL2	1	1	1	1	6.01E-05	1.13E-04	87.16
HM13	1	1	1	1	6.01E-05	1.13E-04	87.16
SLC4A7	1	1	1	1	6.01E-05	1.13E-04	87.16
PCCB	1	1	1	1	6.01E-05	1.13E-04	87.16
RBM6	1	1	1	1	6.01E-05	1.13E-04	87.16
RICTOR	1	1	1	1	6.01E-05	1.13E-04	87.16
NSD1	1	1	1	1	6.01E-05	1.13E-04	87.16
GPC2	1	1	1	1	6.01E-05	1.13E-04	87.16
POT1	1	1	1	1	6.01E-05	1.13E-04	87.16
RNF216	1	1	1	1	6.01E-05	1.13E-04	87.16
PAG1	1	1	1	1	6.01E-05	1.13E-04	87.16
RAD54B	1	1	1	1	6.01E-05	1.13E-04	87.16
SQLE	1	1	1	1	6.01E-05	1.13E-04	87.16
HSF1	1	1	1	1	6.01E-05	1.13E-04	87.16
SCAI	1	1	1	1	6.01E-05	1.13E-04	87.16
SPTAN1	1	1	1	1	6.01E-05	1.13E-04	87.16
RABEP1	2	3	2	1	1.2E-04	3.38E-04	180.74
KCNA3	1	2	2	1	6.01E-05	2.25E-04	274.32
SLC39A11	1	2	1	2	6.01E-05	2.25E-04	274.32
SEPT9	1	2	2	1	6.01E-05	2.25E-04	274.32
GSK3B	1	2	1	2	6.01E-05	2.25E-04	274.32
AP3B1	1	2	1	2	6.01E-05	2.25E-04	274.32
DOP1A	1	2	1	1	6.01E-05	2.25E-04	274.32
STAG3	1	2	1	1	6.01E-05	2.25E-04	274.32
VCL	1	2	1	2	6.02E-05	2.25E-04	274.32
BOLA2-SMG1P6	1	2	2	1	6.01E-05	2.25E-04	274.32
UTP4	1	2	1	1	6.01E-05	2.25E-04	274.32
NARF	1	2	1	2	6.01E-05	2.25E-04	274.32
BABAM2	1	2	1	2	6.01E-05	2.25E-04	274.32
STAT5B	2	5	2	2	1.2E-04	5.63E-04	367.91

FOXK2	1	3	1	2	6.01E-05	3.38E-04	461.49
SMG1P6	1	3	2	1	6.01E-05	3.38E-04	461.49
SMG1P5	1	3	1	2	6.01E-05	3.38E-04	461.49

***** Only showing genes that were identified at least once in both groups in any given pairwise comparison

Supplemental Table 5: Clonal percentages by subset and infection stage

Stage	Subset*	Total unique sites	Clonal sites	% clonal
Acute	CD69-	282	4	1.42
	CD69+	177	0	0.00
	GC-Tfh	213	1	0.47
	Naive	9	0	0.00
	Tcm	144	4	2.78
	Tem	153	3	1.96
	Temra	4	0	0.00
Chronic	CD69-	797	76	9.54
	CD69+	190	8	4.21
	GC-Tfh	368	22	5.98
	Naive	38	6	15.79
	Tcm	112	8	7.14
	Tem	156	10	6.41
	Temra	5	1	20.00
ART	CD69- Tcm	48	5	10.42
	CD69- Tem	266	16	6.02
	HLA-DR- CD69-	225	17	7.56
	HLA-DR- CD69+	259	17	6.56
	HLA-DR- GC-Tfh	88	6	6.82
	Naive	6	0	0.00

* Naïve subsets were pooled between tissue and blood. Tissue subsets for acute and chronic individuals were pooled between inguinal and cervical lymph nodes.
